# Phytate metabolism is mediated by microbial cross-feeding in the gut microbiota

**DOI:** 10.1038/s41564-024-01698-7

**Published:** 2024-06-10

**Authors:** Willem M. De Vos, Minh Nguyen Trung, Mark Davids, Guizhen Liu, Melany Rios Morales, Henning Jessen, Dorothea Fiedler, Max Nieuwdorp, Thi Phuong Nam Bui

**Affiliations:** 1 Laboratory of Microbiology, Wageningen University, Wageningen, The Netherlands; 2 Leibniz-Forschungsinstitut für Molekulare Pharmakologie, Berlin, Germany; 3 Institute of Chemistry, Humboldt-Universität zu Berlin, Berlin, Germany; 4 Departments of Internal and Experimental Vascular Medicine, Amsterdam University Medical Centers, Location AMC, Amsterdam, the Netherlands; 5 Institute of Organic Chemistry, University of Freiburg, Freiburg, Germany; 6 Department of Surgery, Spaarne Hospital, Hoofddorp, the Netherlands

## Abstract

Dietary intake of phytate has various reported health benefits. Previous work showed that the gut microbiota can convert phytate to short-chain fatty acids (SCFAs), but the microbial species and metabolic pathway are unclear. Here, we identified *Mitsuokella jalaludinii* as an efficient phytate degrader, which works synergistically with *Anaerostipes rhamnosivorans* to produce the SCFA propionate. Analysis of published human gut taxonomic profiles revealed that *Mitsuokella* spp., in particular *M. jalaludinii*, are prevalent in human gut microbiomes. NMR spectroscopy using ^13^C-isotope labelling, metabolomic and transcriptomic analyses identified a complete phytate degradation pathway in *M. jalaludinii*, including production of the intermediate Ins(2)P/*myo*-inositol. The major end product, 3-hydroxypropionate, was converted into propionate via a synergistic interaction with *Anaerostipes rhamnosivorans* both in vitro and in mice. Upon [^13^C_6_]phytate administration, various ^13^C-labelled components were detected in mouse cecum in contrast with the absence of [^13^C_6_] InsPs or [^13^C_6_]-*myo*-inositol in plasma. Caco-2 cells incubated with co-culture supernatants exhibited improved intestinal barrier integrity. These results suggest that the microbiome plays a major role in the metabolism of this phytochemical and that its fermentation to propionate by *M. jalaludinii* and *A. rhamnosivorans* may contribute to phytate-driven health benefits.

## Introduction

Understanding the interaction between diet, the microbiome and the host is key to maintain host well-being^1–2^. The dietary component phytate (*myo*-inositol-1,2,3,4,5,6-hexakisphosphate or InsP_6_) is widely distributed in the plant kingdom and especially abundant in wheat, rice and nuts^3^. The daily intake of this phytochemical is on average approximately 2.5 g phytate per person^3–5^. Phytate has been regarded as an anti-nutrient in animal feed^6^ owing to its strong metal-chelating properties. Yet, to date there is no evidence that phytate intake in human might be problematic due to reduced bioavailability of minerals and other food components. In contrast, health benefits have been reported for the consumption of plant-based diets, including phytate-rich products, like nuts, seeds and unprocessed whole grains^2, 7–8^. Dietary phytate supplementation reportedly promoted epithelial repair resulting in improved barrier function^9^, reduced serum levels of glycated hemoglobin HbA1c and Advanced Glycation End products^10^, improved glucose metabolism^11^, reduced inflammation^12^ and exerted protective effects against colon cancer^13^. Nevertheless, the molecular mechanism by which dietary phytate confers these health benefits is largely unknown.

Phytate not only derives from our diet but is also one of the most abundant inositol phosphates (InsPs) in mammalian cells and is synthesized as part of the intracellular metabolism of *myo*-inositol^14–16^. Phytate is involved in a variety of key physiological processes including glucose metabolism, insulin signaling, cell migration as well as cancer metastasis^16–20^. However, it is not clear whether dietary phytate can enter the systemic circulation and contribute to endogenous inositol polyphosphate biosynthesis. Studies in mice using radioactively labelled dietary phytate indicated that only a small fraction of phytate is reaching the systemic circulation as inositol^21^.

We recently reported that under anaerobic conditions, the human fecal microbiome can fully convert phytate into short-chain fatty acids (SCFAs), including propionate and butyrate^22^, which are well known microbial signaling molecules and strongly associated with host health^23–24^. Nevertheless, the microbes responsible for metabolizing phytate in the human gut are unknown. Only a few *Bifidobacterium, Lactobacillus, Escherichia* and *Bacteroides* spp. have been found to partially convert phytate to lower phosphorylated InsPs (isomers of InsP_3_; InsP_4_ and InsP_5_)^9, 25–29^. Previous efforts to detect and cultivate phytate-degrading microbes from human fecal samples have remained unsuccessful mainly due to the non-optimal growth conditions for strict anaerobes^30^. This contrasts with animal studies that identified phytase-producing *Mitsuokella* spp. in ruminants^31^. *Mitsuokella* spp. are Gram-negative gut anaerobes belonging to the Negativicutes and the rumen isolate *M. multacida* possesses a phytase (an enzyme to break down phytate) located in the outer membrane^32^.

To investigate how dietary phytate is metabolized by human gut bacteria in vitro and in vivo, we here employed a combination of nuclear magnetic resonance (NMR) spectroscopy and stable-isotope labelling of phytate^33^, supported by capillary electrophoresis mass spectrometry (CE-MS) for the detection of inositol phosphates^15^. We identified *Mitsuokella* spp. as efficient and prevalent phytate-degrading bacteria in the human gut whose abundance is strongly associated with ethnicity and host health in large human cohorts. We further characterized the phytate degradation pathway of the most prevalent *M. jalaludinii* using [^13^C_6_]phytate, and transcriptomic and metabolomic analyses. Results revealed the production of the anti-microbial end-product 3-hydroxypropionate that can be efficiently converted by *Anaerostipes rhamnosivorans* in a synergistic interaction to propionate that improved barrier integrity. These results indicate that colonic phytate can be completely converted by a trophic chain involving *M. jalaludinii* and *A. rhamnosivorans* into propionate and acetate that may contribute to dietary phytate-driven health benefits.

## Results

### Identification of *Mitsuokella* spp. as efficient phytate degraders

**Figure 1:**
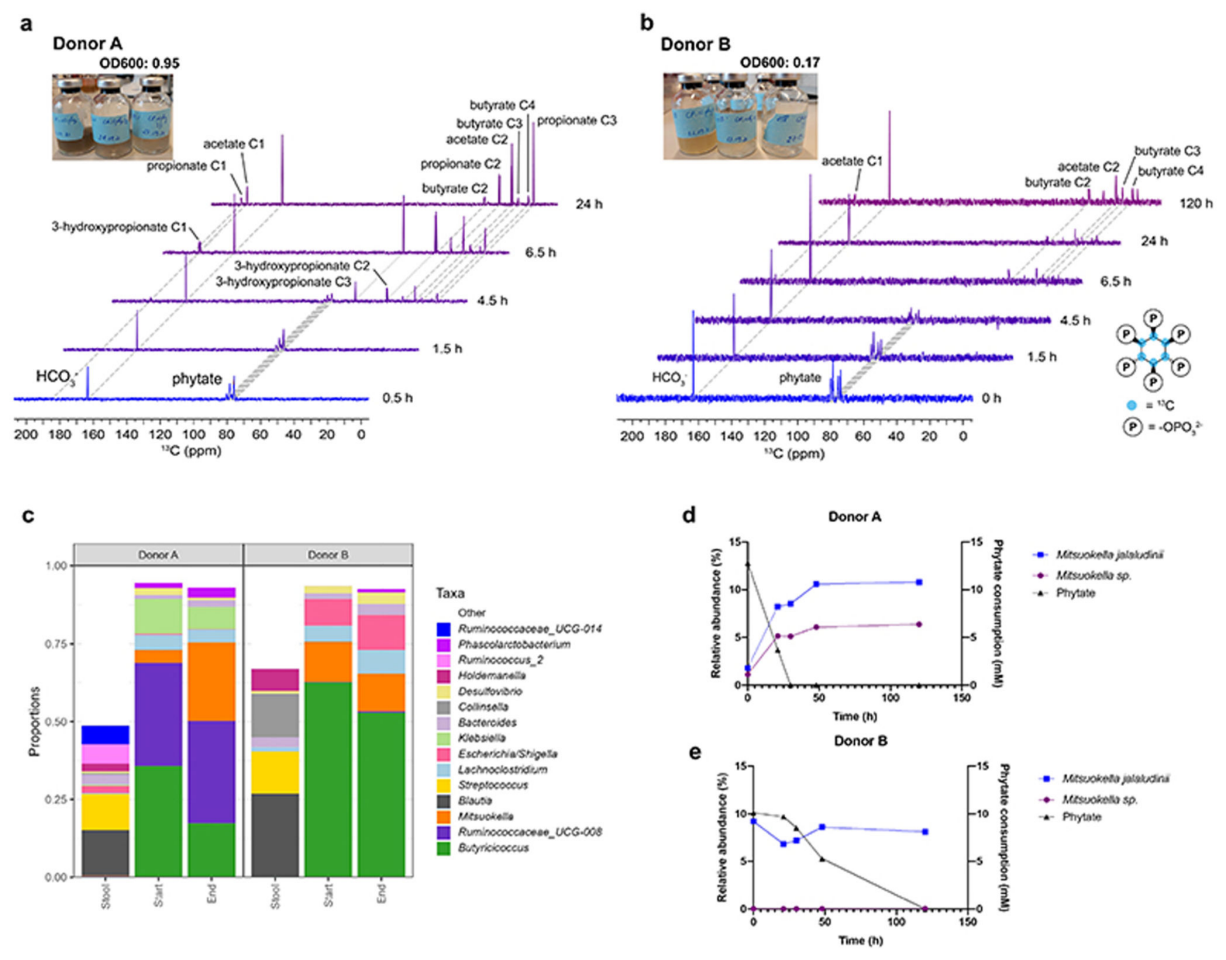
Fecal phytate metabolism and identification of phytate-degrading *Mitsuokella* spp. **a:** ^13^C-NMR spectra of [^13^C_6_]InsP_6_ metabolism by fecal microbiome of donor A during a 24 h incubation. Dashed lines connect NMR signals of identified metabolites and CO_2_. For 3-hydroxypropionate, dotted lines are used for better visibility. The final metabolic products derived from [^13^C_6_]InsP_6_ are [^13^C_2_]acetate, [^13^C_3_]propionate, and [^13^C_4_]butyrate. [^13^C_3_]3-hydroxypropionate accumulates transiently between 4.5 - 6.5 h. **b**: ^13^C-NMR spectra of [^13^C_6_]InsP_6_ metabolism by the fecal microbiome of donor B during up to 5 days incubation. The final metabolic products derived from [^13^C_6_]InsP_6_ are [^13^C_2_]acetate, and [^13^C_4_]butyrate. Structure of [13C6]InsP6 shown in the lower right. **c**: Microbial composition of fecal samples, the initial time points and end time points (5 days) of the third transfer of phytate enrichments from donor A and B (n=1 biological replicate per donor). Top 15 abundant taxonomic groups in these samples are shown in colours and the rest is shown as ‘other’ in white. **d**: Changes of relative abundance of *Mitsuokella jalaludinii* and *Mitsuokella* spp. during phytate incubation and phytate consumption (right vertical axis) in the third transfer of the fecal enrichment from donor A. **e**: Changes of relative abundance of *Mitsuokella jalaludinii* and *Mitsuokella* spp. during phytate incubation and phytate consumption (right vertical axis) in the third transfer of the fecal enrichment from donor B.

To gain a better understanding which bacteria are involved in the rapid degradation of phytate, we incubated fresh fecal samples from two healthy donors (A and B) in a medium supplemented with [^13^C_6_]InsP_6_ as the sole carbon and energy source. The supernatants from [^13^C_6_]InsP_6_ enrichments were collected over time and used for the analysis of ^13^C-labelled components by ^13^C-NMR. In parallel, non-labelled phytate fecal enrichments were repeatedly transferred to fresh phytate media to further enrich for phytate degrading microbes. We observed that the fecal microbiome of participant A metabolized [^13^C_6_]InsP_6_ within a few hours to [^13^C_2_]acetate and [^13^C_3_]3-hydroxypropionate, which was subsequently converted to [^13^C_3_]propionate after 24h (Fig. 1a). The fecal microbiome of donor B slowly used [^13^C_6_]InsP_6_ to produce [^13^C_4_]butyrate and [^13^C_2_]acetate without the formation of any other intermediates (Fig. 1b). This difference in phytate metabolism was also reflected by the reduced microbial density in the first three non-labelled phytate enrichment transfers of donor B compared with that of donor A (Fig. 1a,b).

To identify the phytate-degrading microbes, genomic DNA was isolated from the third non-labelled phytate enrichment and subjected to 16S ribosomal RNA gene amplicon sequencing. We observed two different microbial communities enriched from faecal microbiomes, with *Ruminococcaceae, Mitsuokella* and *Butyricicoccus* as the most abundant taxa in donor A and *Butyricicoccus, Mitsuokella* and *E.coli/Shigella* as the most abundant taxa in donor B (Fig. 1c). This may explain the different phytate fermentation patterns where 3-hydroxypropionate was formed as intermediate in donor A while donor B showed no intermediate. At the end of the phytate incubation, the relative abundance of most species decreased, whereas that of *Mitsuokella* spp. increased in both enrichments up to 10 % of total microbiome when phytate was used, with *M. jalaludinii* as the most dominant species (Fig. 1d,e). Of note, the relative abundance of *Mitsuokella* increased gradually during the first 48 h, which was in line with a rapid degradation of phytate within 30 h for donor A (Fig. 1d) whereas the relative abundance of *Mitsuokella* only increased after 24 h due to the limited degradation of phytate during the first 24 h for donor B (Fig. 1e). Overall, these results suggest that *M. jalaludinii* is involved in phytate degradation in the human gut.

### *Mitsuokella* as prevalent taxon in the human intestine and its health relation

**Figure 2:**
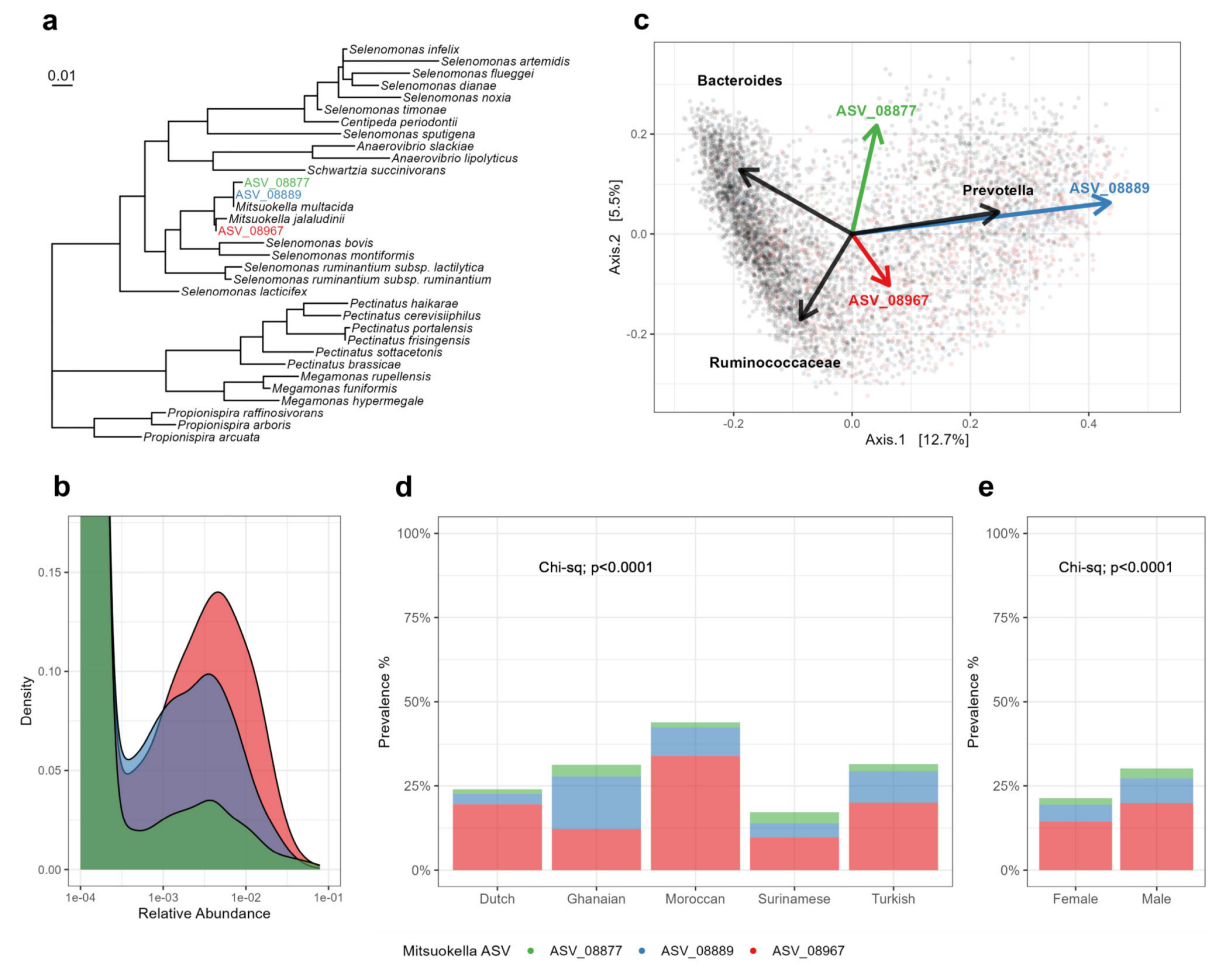
Phylogeny, ecology and distribution of *Mitsuokella* spp. in a human cohort study. **a:** Phylogenetic tree showing the phylogenetic placement of thee prevalent *Mitsuokella* ASVs (ASV_08877, ASV_08889 and ASV_08967) in Selenomonadales cluster in an all-species living tree. Based on the tree, ASV_08877 (green) and ASV_08889 (blue) are highly similar to *M. multacida* while ASV_08967 (red) is close to *M. jalaludinii*. **b**: Density plot of the relative abundance of 3 these different ASVs in 6039 participants of HELIUS cohort indicates that if present, *Mitsuokella* constitutes 0.01 to 10 % of the bacterial community. **c**: Principal coordinate analysis of microbiome composition of the HELIUS cohort. Arrows indicate weighted average scores of the three prevalent *Mitsuokella* ASVs which are strongly associated with *Prevotella* enterotype. **d**: Distribution of the 3 prevalent *Mitsuokella* ASVs in the 5 major ethnic groups including Dutch (n=1615), Ghanaian (n=614), Moroccan (n=830), Surinamese (n=2380) and Turkish (n=585), of the HELIUS cohort, with ASV_08967 (*M. jalaludinii*) as the most abundant ASV except the Ghanaian ethnicity group. **e**: Distribution *Mitsuokella* ASVs between females (n=3204) and males (n=2820).

To further investigate the prevalence of *Mitsuokella* in the general population and determine its ecological niche, we analyzed the microbiome of 6039 Amsterdam-located subjects in HELIUS cohort with different ethnicities^34–35^. We found three amplicon sequence variants (ASV) of *Mitsuokella* which represented 89 % of all *Mitsuokella* counts. These sequence variants were highly similar to those belonging to *Mitsuokella multacida* or *Mitsuokella jalaludinii* (Fig. 2a). A total of 1542 out of 6039 subjects were positive for one of these ASVs, indicating the moderate prevalence of *Mitsuokella* spp. that showed relative abundances between 0.01 to 10 % (Fig. 2b). *Mitsuokella* spp. are closely related to known fiber-degrading species, including ruminal *Selenomonas* spp. (Fig. 2a)^36^, one of which has been reported to contain an active periplasmic phytase^31–32^. *Mitsuokella* spp. were also strongly associated with the *Prevotella* enterotype (Fig. 2c), which has been linked with high fiber intake and health status^37–38^. The prevalence of different *Mitsuokella* spp. varied slightly among the ethnicities, but most participants harbored *M. jalaludinii* with highest prevalence in males (Fig. 2d,e). To verify its functionality, we isolated a dominant *Mitsuokella jalaludinii* strain from donor A and found it to grow fast on phytate and its genome sequence to be highly similar (an average nucleotide identity of 97.3 %) to that of the type-strain *M. jalaludinii* DSM13811^T^ with greatly identical (95-100 %) phytate degradation pathway genes (Sup. Fig. 1 and Table S1). Hence, this confirms that *M. jalaludinii* is prevalent in humans. It was recently reported that the abundance of fecal *Mitsuokella* spp. was significantly lower in cardiometabolic patients compared to the healthy population^39^. This prompted us to further study the role of *M. jalaludinii* in phytate degradation and its contribution to host health.

### Elucidation of phytate degradation pathway by *Mitsuokella jalaludinii*

**Figure 3:**
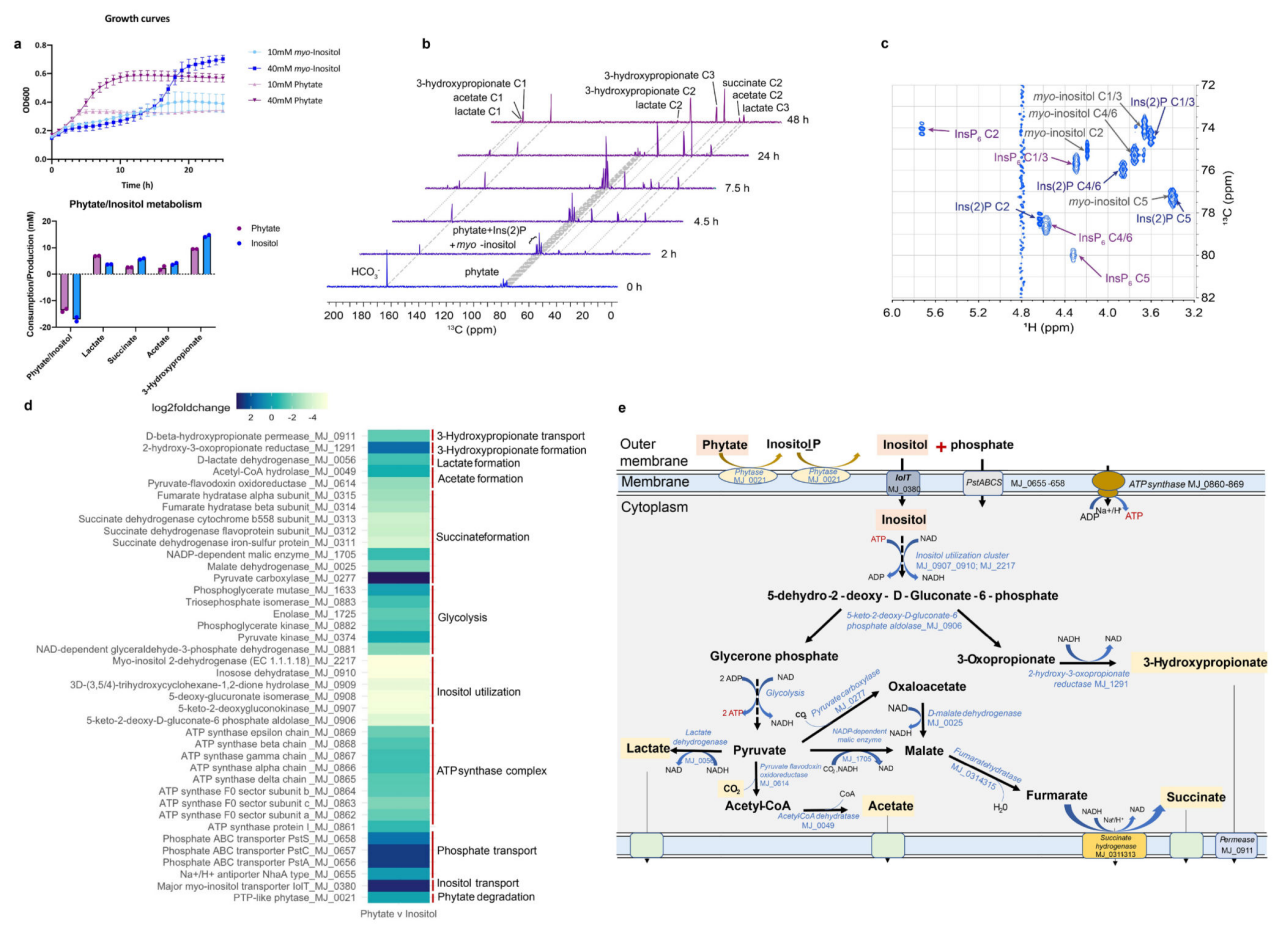
Elucidation of phytate degradation pathway in *Mitsuokella jalaludinii* DSM13811^T^. **a:** Faster growth of *M. jalaludinii* in 10 mM and 40 mM phytate as compared to growth in 10 mM and 40 mM myo-inositol (top), while similar metabolite production (bottom) was observed. Data are presented as mean values +/- SD (n=3 biological replicates for 10 mM, 2 for 40 mM and 2 for metabolite measurements). **b**: ^13^C-NMR analysis shows rapid [^13^C_6_]phytate degradation with [^13^C_6_]Ins(2)P and [^13^C_6_]Inositol as intermediates and [^13^C_3_]3-hydroxypropionate, [^13^C_3_]lactate, [^13^C_4_]succinate, and [^13^C_2_]acetate as end metabolites. **c**: BIRD-{^1^H,^13^C}HMQC spectrum of the *M. jalaludinii* culture sample at 7.5 h. The region in which inositol polyphosphate signals are commonly found is shown. The mixture contains the starting material [^13^C_6_]InsP_6_ (purple arrows) but also the dephosphorylation products Ins(2)P (blue arrows) and *myo*-inositol (grey arrows). The residual solvent signal (HDO) appears at 4.8 ppm. Each detectable signal is annotated with the corresponding substance name and position of the inositol ring. Note that each of the detected substances exhibit four NMR signals due to the C_s_ symmetry of the six-membered inositol ring and therefore, positions C1 and C3, and positions C4 and C6 are magnetically equivalent. **d**: Log_2_(fold change) of differential expression of genes involved in phytate degradation; inositol and phosphate uptake and inositol metabolic genes comparing between phytate and inositol conditions (Table S2). Predicted functional groups of these genes are indicated on the right. **e**: Reconstruction of the entire phytate degradation pathway based on genomic, transcriptomic and metabolomic analyses. The genes have been identified a genome *M. jalaludinii* DSM13811^T^ from GenBank (BioProject PRJNA223472) and re-annotating the genome in RAST (Table S3).

We used *M. jalaludinii* DSM13811^T 40^ for proof-of-concept experiments and cultured it in a medium containing phytate or *myo*-inositol as the sole energy and carbon source. *M. jalaludinii* grew rapidly in phytate with a doubling time of 3.4 h while the doubling time with *myo*-inositol was 7 h (Fig. 3a). The metabolite production was similar between both conditions. On the basis of the observed stoichiometry of phytate conversion, we propose the net theoretical fermentative equation as: 1 phytate + 6 H_2_0 → 1 3-hydroxypropionate + 0.4 lactate + 0.2 succinate + 0.2 acetate + 0.6 CO_2_ + 6 phosphate. Of note, the formation of CO_2_ was not determined due to the pre-existing bicarbonate in the medium but predicted on the basis of carbon and redox recovery.

**Extended Figure 1:**
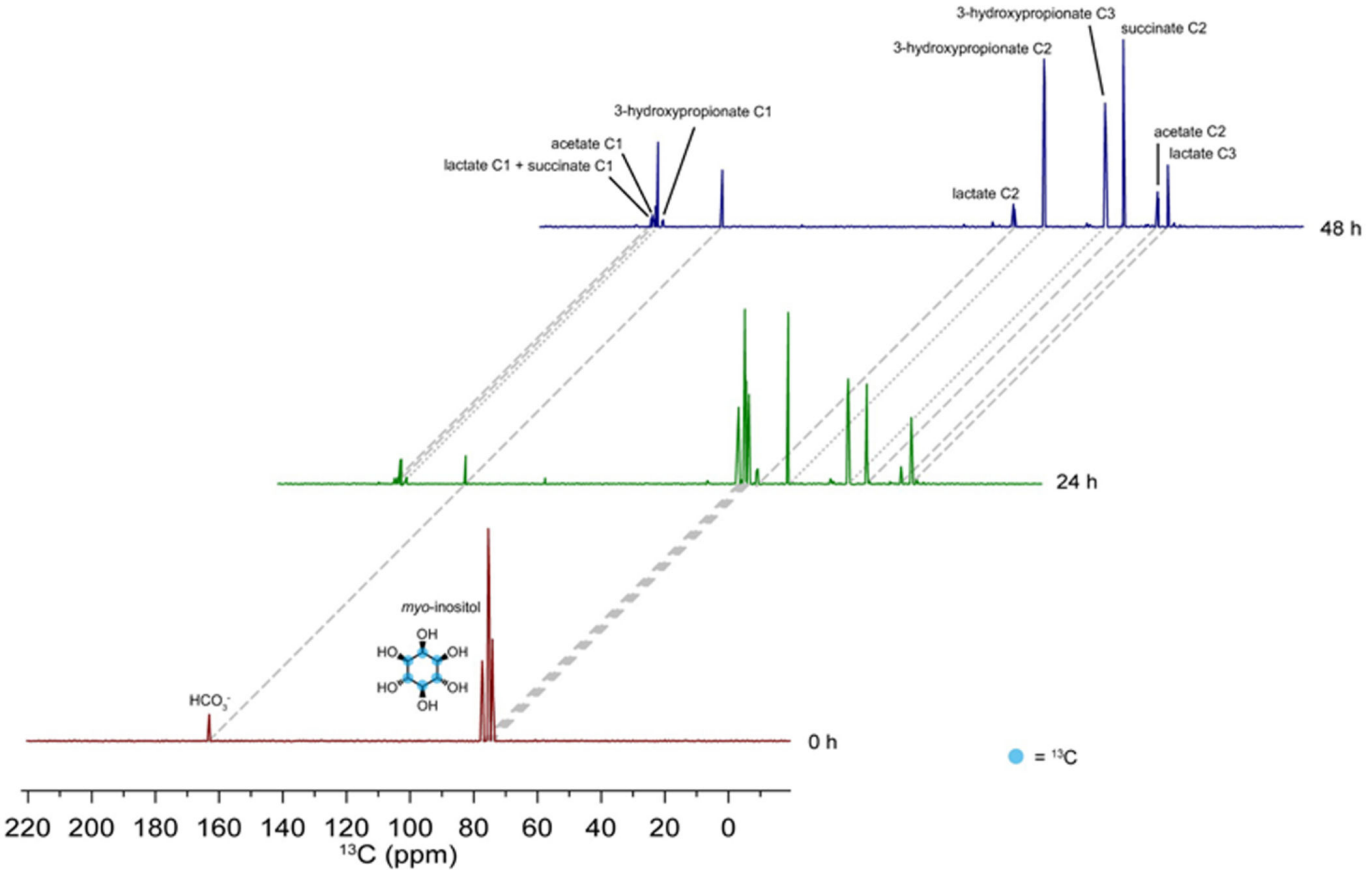
Stable-isotope analysis of *myo*-inositol metabolism in *M. jalajudinii*. ^13^C-NMR analysis shows rapid [^13^C_6_]*myo*-inositol conversion by *M. jalaludinii* DSM13811^T^ to [^13^C_3_]3-hydroxypropionate, [^13^C_3_]lactate, [^13^C_4_], and [^13^C_2_]acetate after 24 h and 48 h incubation. incubation. Raw NMR data are provided as supplementary data (Bui_NMR_data 4).

To unambiguously identify the pathway, its intermediates and all end metabolites of phytate degradation, *M. jalaludinii* was grown in bicarbonate buffered medium containing either [^13^C_6_]phytate or [^13^C_6_]*myo*-inositol as the sole energy and carbon source. As observed by 1D-NMR and 2D-NMR spectroscopy^33^, *M. jalaludinii* converted [^13^C_6_]phytate rapidly to various metabolites with [^13^C_3_]3-hydroxypropionate, [^13^C_3_]lactate and [^13^C_4_]succinate as major end metabolites (Fig. 3b). We identified the accumulation of *myo-*inositol-2-monophosphate (Ins(2)P) and *myo*-inositol during the first 7.5 h via 2D-NMR (Fig. 3c), suggesting that these are intermediates of phytate degradation. The capacity to use *myo*-inositol was confirmed by the growth of *M. jalaludinii* and conversion of [^13^C_6_]*myo*-inositol to [^13^C_3_]3-hydroxypropionate, [^13^C_3_]lactate and [^13^C_4_]succinate (Extended Fig. 1). The production of 3-hydroxypropionate from phytate confirmed our previous hypothesis that *Mitsuokella* spp. were responsible for the efficient fecal phytate degradation in which 3-hydroxypropionate was detected as intermediate (Fig. 1a).

**Extended Figure 2:**
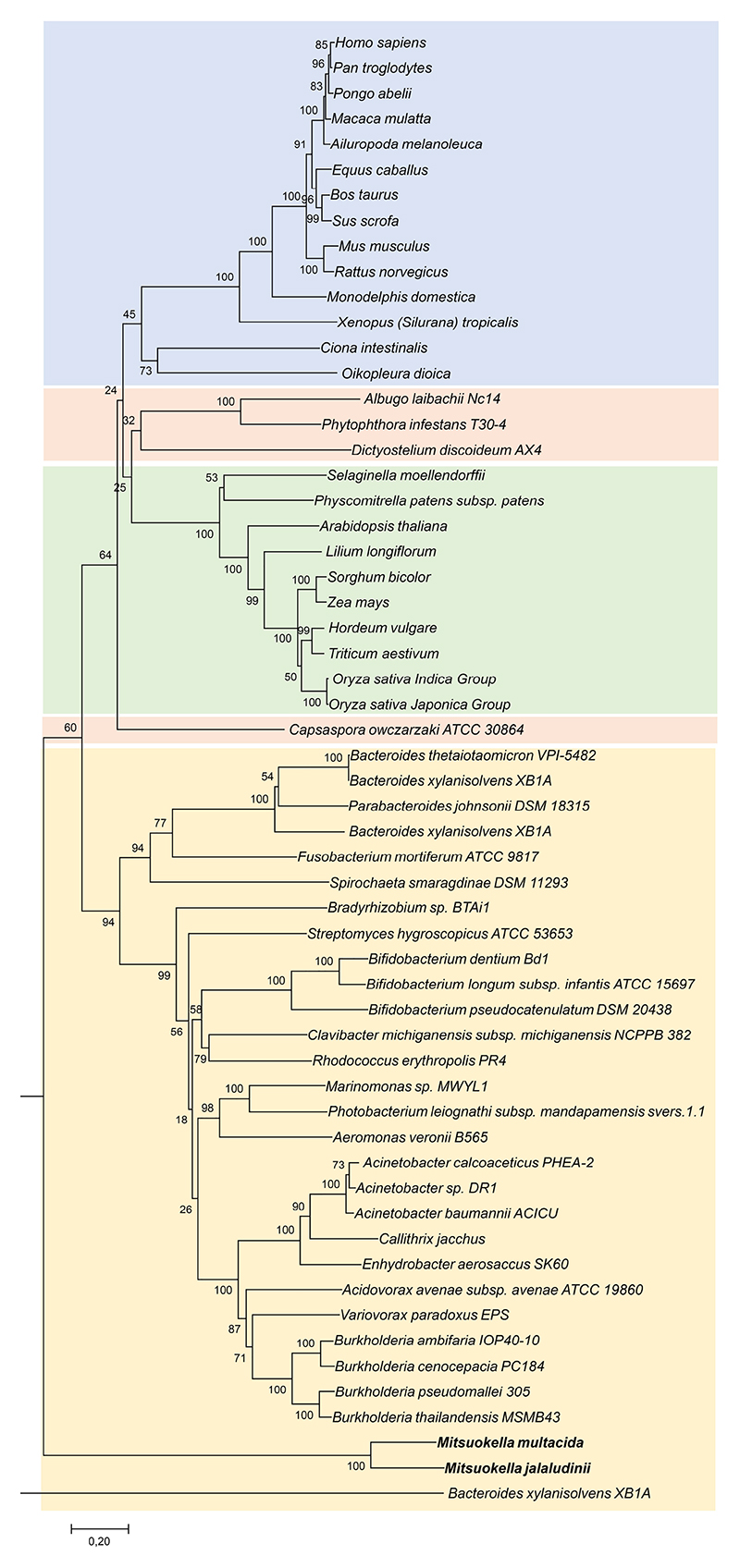
Phylogeny of phytases. A phylogenetic tree derived from the alignment of predicted phytases from *M. jalaludinii* and *M. multacida* (bold) and MINPP protein representatives from different kingdoms of life was constructed using the Maximum Likelihood algorithm. The different kingdoms have been assigned the following background colours: Animal, blue; plants, green; protists. orange; bacterial, yellow. Bar, 20 % sequence divergence.

To further identify genes of *M. jalaludinii* involved in phytate metabolism, we analyzed transcriptomic profiles of cells grown in either phytate or *myo*-inositol (Sup. Fig. 2; Table S2). This analysis showed that the expression of the genes for an inositol transporter (MJ_0380) and a high-affinity phosphate transport system PstSCAB^41^ (MJ_0656-0658) were eight-fold increased during the growth on phytate, as well as the electron transport chain by a succinate dehydrogenase membrane complex^42^ (MJ_0311-0313) and ATP synthase (MJ_0861-0869). We also identified induced expression of 2-hydroxy-3-oxopropionate reductase (MJ_1291) and a 3-hydroxypropionate permease (MJ_0911) involved in 3-hydoxypropionate production and transport, respectively. Remarkably, the gene encodes a periplasmic phytase (MJ_0021) was found to be constitutively expressed (Fig. 3d). Evolutionary analysis showed that the encoded phytase was only distantly related to other microbial and human phytases (Extended Fig. 2), which may imply its unique capacity to readily dephosphorylate phytate to inositol (Fig. 3b-c). In addition, genes involved in acetate, lactate and succinate, 3-hydroxypropionate production, glycolysis and ATP synthase were identified, all of which except a pyruvate carboxylase were expressed constitutively (Fig. 3d). The metabolomic and transcriptomic analyses imply that the faster growth of *M. jalaludinii* on phytate as compared to *myo*-inositol may probably be due to the increased activity of the inositol transporter. On the basis of the NMR and transcriptomic analyses, the entire metabolic phytate degradation pathway was reconstructed (Fig. 3e). The observation that a monoculture of *M. jalaludinii* mainly produced succinate and 3-hydroxypropionate and not the canonical short chain fatty acids from phytate suggests that there are metabolic interactions between *Mitsuokella* with other commensal bacteria in the human colon.

### In vitro synergy between *M. jalaludinii* and *A. rhamnosivorans* in phytate

**Extended Figure 3:**
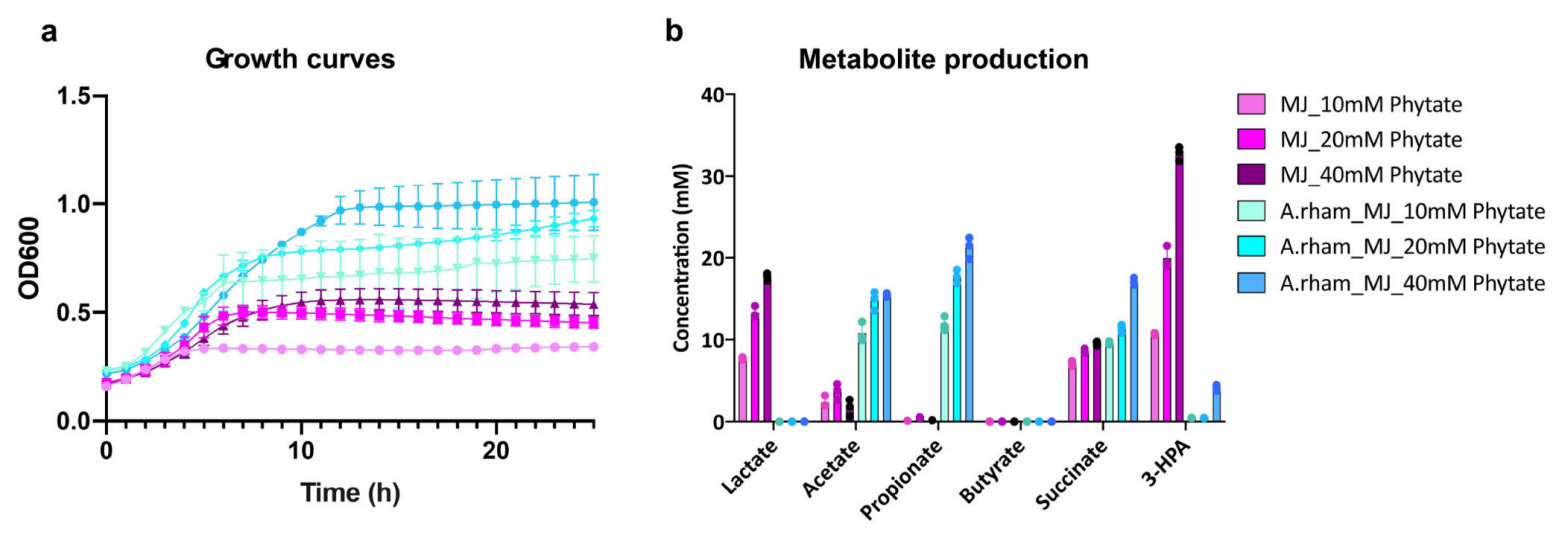
Concentration independent synergy between *M. jalaludinii* and *A. rhamnosivorans* for phytate breakdown. **a**: Growth curves of co-cultures (blue) of *M. jalaludinii* a nd *A. rhamnosivorans* and monocultures (pink) of *M. jalaludinii* in 10 mM, 20 mM, and 40 mM phytate during 24 h incubation. **b**: End metabolite production of the co-cultures and monocultures of cocultures (blue) of *M. jalaludinii* and *A. rhamnosivorans* and monocultures (pink) of *M. jalaludinii* in 10 mM, 20 mM, and 40 mM phytate after 24 h incubation. Data are presented as mean values +/- SD (n=3 biological replicates).

**Extended Figure 4:**
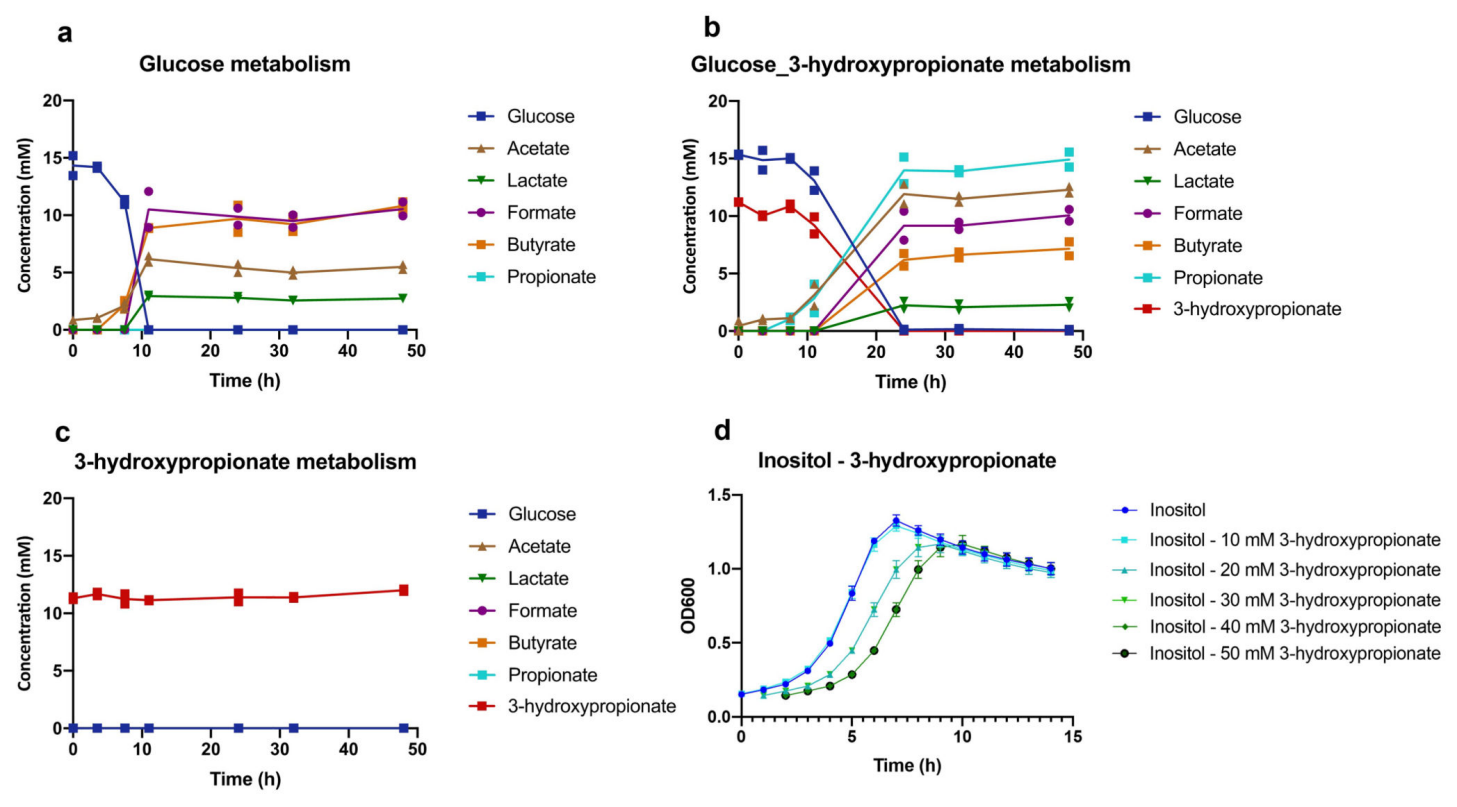
3-hydroxypropionate metabolism by *A. rhamnosivorans*. *A. rhamnosivorans* was found to co-metabolize 3-hydroxypropionate but failed to use it as sole energy and carbon. Fermentation profiles are shown of *A. rhamnosivorans* incubated in glucose (**a**); glucose and 3-hydroxypropionate (**b**); 3-hydroxypropionate (**c**) and *myo-*inositol plus 3-hydroxypropionate (HP) (**d**) by *Anaerostipes rhamnosivorans*. All experiment were performed in biological duplicate. Data are presented as mean values (n=2 biological replicates for a, b, c) or mean values +/- SD (n=4 biological replicates for d).

**Figure 4:**
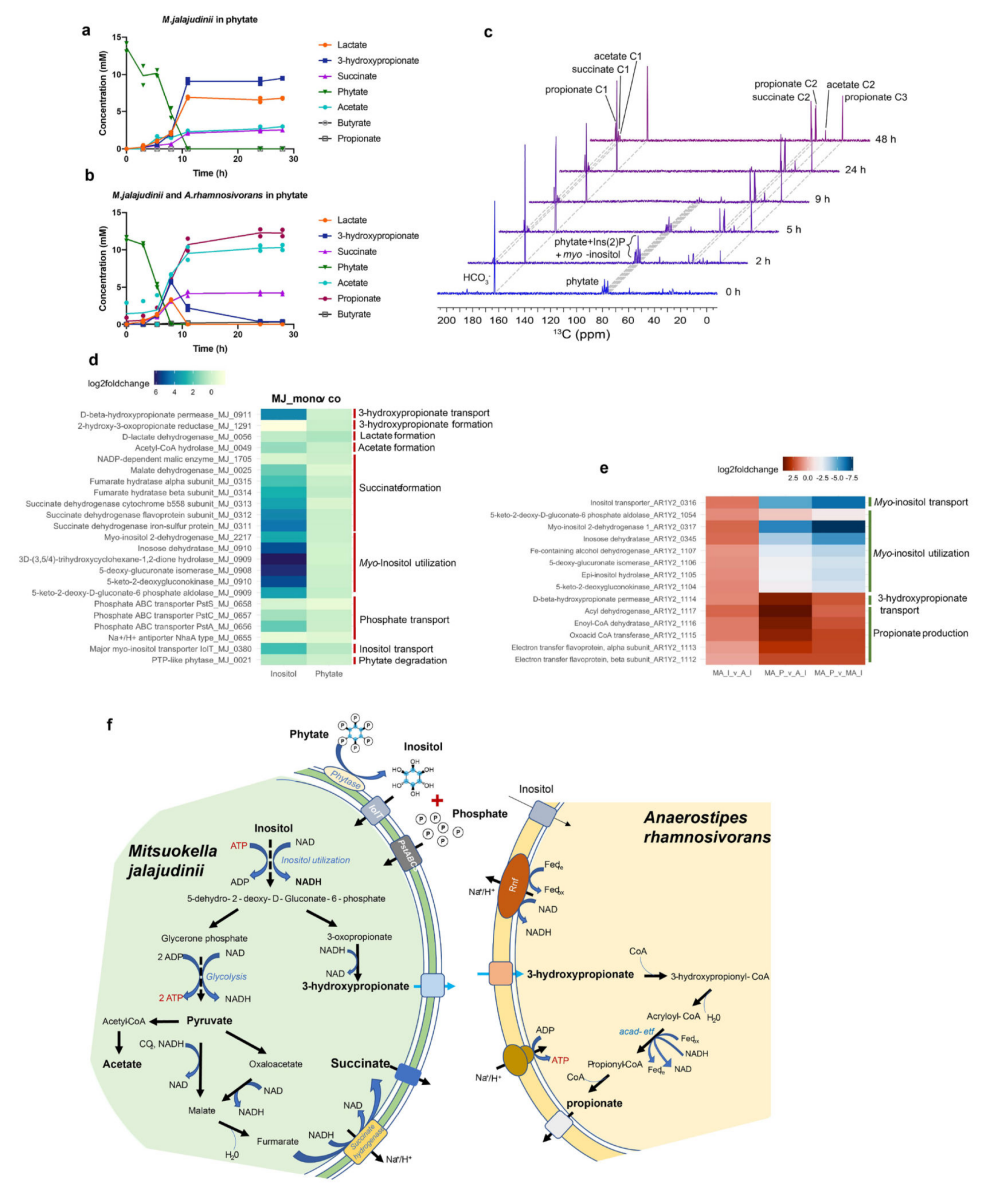
Mechanistic insight into the synergy between *M. jalaludinii* and *A. rhamnosivorans* in phytate degradation. **a-b:** Metabolite production and substrate consumption by monoculture of *M. jalaludinii* (**a**) and coculture of *M. jalaludinii* and *A. rhamnosivorans* (**b**) in phytate (n=2 biological replicates). Data are presented as mean values of 2 data points. **c**: ^13^C-NMR spectra of [^13^C_6_]InsP_6_ metabolism by coculture of *M. jalaludinii* and *A. rhamnosivorans* during a 48 h incubation. Dashed lines connect NMR signals of identified metabolites and CO_2_. Dotted lines are used for 3-hydroxypropionate to provide better visibility_._ The final metabolic products derived from [^13^C_6_]InsP_6_ are [^13^C_2_]acetate, [^13^C_3_]propionate, [^13^C_3_]lactate, [^13^C_3_]3-hydroxypropionate, [^13^C_4_]butyrate, and [^13^C_4_]succinate. [^13^C_4_]succinate is again formed as a mixture with partially or non-^13^C-labeled succinate. The same InsP intermediates can be observed between 2 - 7.5 h as in the monoculture (Fig. 4b). **d**: *M. jalaludinii* (MJ): Log_2_(fold change) of differential expression of genes involved in phytate degradation; inositol and phosphate uptake and inositol metabolic genes comparing between monoculture and coculture (Table S4-5). The functions of these genes are indicated on the right. **e**: *A. rhamnosivorans*: Log_2_(fold change) of differential expression of genes involved in inositol uptake, inositol metabolic genes, propionate production and 3-hydroxypropionate transport comparing between MA_I (coculture in *myo*-inositol) versus A_I (monoculture in *myo*-inositol); MA_P (coculture in phytate) versus A_I (monoculture in *myo*-inositol); MA_P (coculture in phytate) versus MA_I (coculture in *myo*-inositol) (Table S6-8). Predicted functional groups of these genes are indicated on the right. The transcriptomic analysis was performed in biological triplicates. **f**: Reconstruction of synergistic interaction between M. *jalaludinii* and *A. rhamnosivorans* in phyate. *M. jalaludinii* uses periplasmic phytase to dephosphorylate phytate rapidly to Ins(2)P and inositol, which can subsequently be taken up and used to produce succinate, acetate, lactate and 3-hydroxypropionate. The produced 3-hydroxypropionate is released into the medium and inhibits *myo*-inositol uptake by *A. rhamnosivorans. A. rhamnosivorans*, in turn, metabolizes 3-hydroxypropionate via a 3-hydroxypropionate scavenging pathway to produce propionate, conserving energy by coupling the electrochemical gradient generated via a membrane bound Rnf complex, with ATP synthesis.

We previously showed that supplementation of *A. rhamnosivorans* in fecal phytate enrichments increased propionate formation^22^. Hence, we investigated the potential synergy between *M. jalaludinii* and *A. rhamnosivorans* in phytate degradation. We observed that the co-cultures reached higher cell density compared to monocultures of *M. jalaludinii* independent of the phytate concentration (Extended Fig. 3a). We detected propionate and acetate in the cocultures, while 3-hydroxypropionate and lactate only accumulated in the *M. jalaludinii* monoculture (Extended Fig. 3b). This indicates a potential synergy between these two bacteria and hence we compared the phytate metabolism in a monoculture of *M. jalaludinii* versus a coculture of *M. jalaludinii* and *A. rhamnosivorans* using both non-labelled and ^13^C_6_-isotopomer of phytate. We observed that 3-hydroxypropionate was first produced and subsequently converted to propionate after 8 h in the coculture (Fig. 4b-c), while 3-hydroxypropionate accumulated in the monoculture of *M. jalajudinnii* (Fig. 4a), suggesting a trophic chain involving 3-hydroxypropionate as an intermediate. We found that *A. rhamnosivorans* was indeed able to convert 3-hydroxypropionate to propionate but only in presence of glucose as carbon source, indicating a co-metabolic conversion (Extended Fig. 4a-c). Of note, we observed that the growth of *A. rhamnosivorans* was inhibited by 3-hydroxypropionate in a dose-dependent way (Extended Fig. 4d). These results imply that the synergy between the two intestinal bacteria was mainly via 3-hydropropionate interspecies transfer. Furthermore, ^13^C-NMR analysis confirmed ^13^C-labelled propionate, succinate and acetate as end metabolites from [^13^C_6_]InsP_6_ in the coculture (Fig. 4c), with [^13^C_6_]Ins(2)P and [^13^C_6_]Inositol as intermediates. The commonalities between the monocultures and cocultures indicate the limited influence of *A. rhamnosivorans* on phytate dephosphorylation by *M. jalaludinii* (Fig. 4c and Sup. Fig. 3).

**Extended Figure 5:**
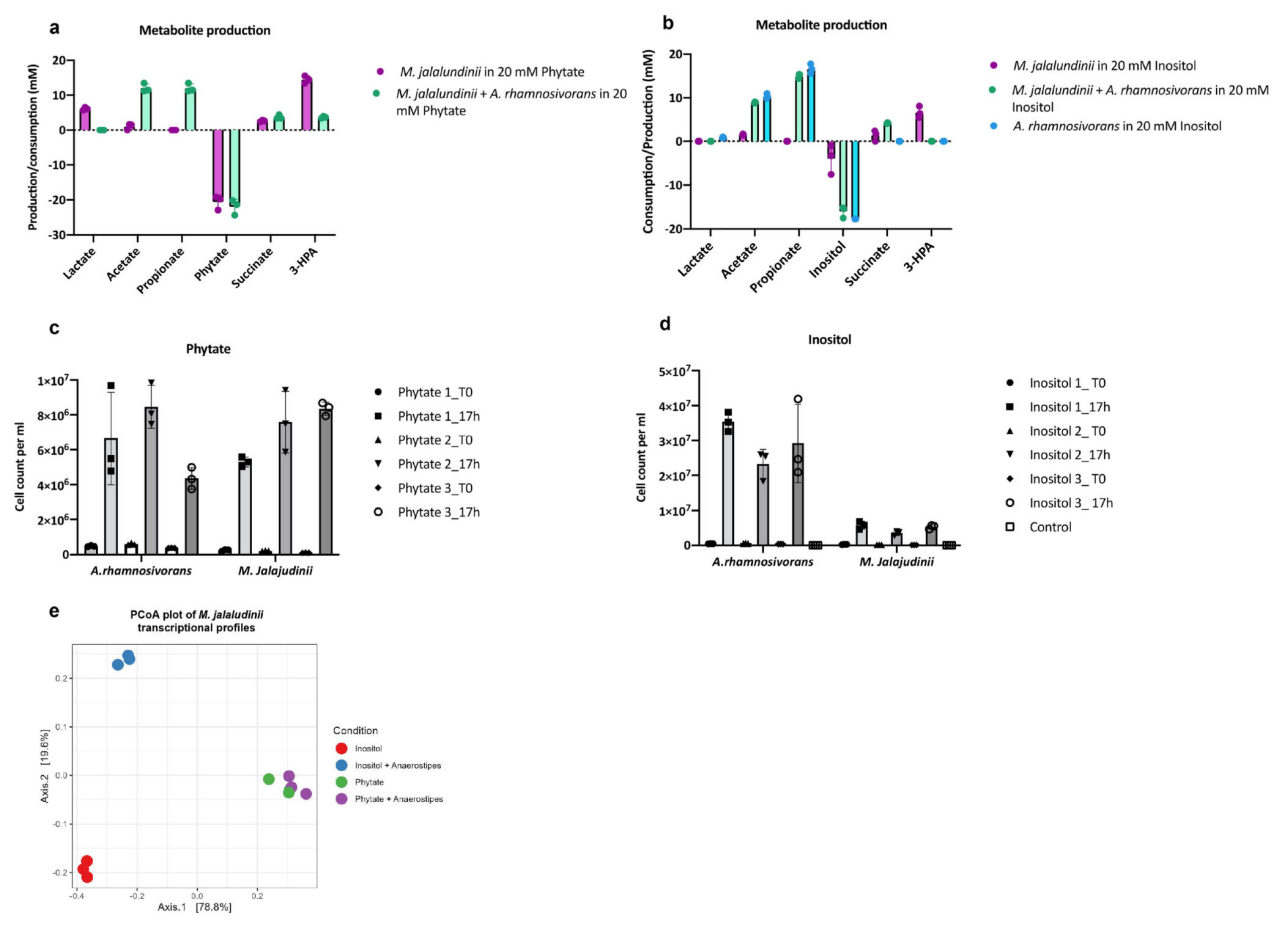
Transcriptome analysis reveals synergy study *M. jalaludinii* and *A. rhamnosivorans*. Metabolite production and substrate consumption of phytate (**a**) and *myo*-inositol (**b**) by monocultures and co-cultures of *M. jalaludinii* and *A. rhamnosivorans*. Cell numbers of *M. jalaludinii* and *A. rhamnosivorans* quantified by qPCR in the coculture on phytate (**c**) and *myo*-inositol (**d**). **e**: Principal coordination plot of the transcriptional profiles obtained by RNAseq of *M. jalaludinii* when grown on phytate (green/purple) or *myo*-inositol (red/blue) in the presence (blue/purple) or absence (red/green) of *A. rhamnosivorans* (Table S2-7). Ordination was based on centered log transformed counts and Euclidean distance. The experiment was performed in biological triplicate. Data are presented as mean values +/- SD (n=3 biological replicates).

**Extended Figure 6:**
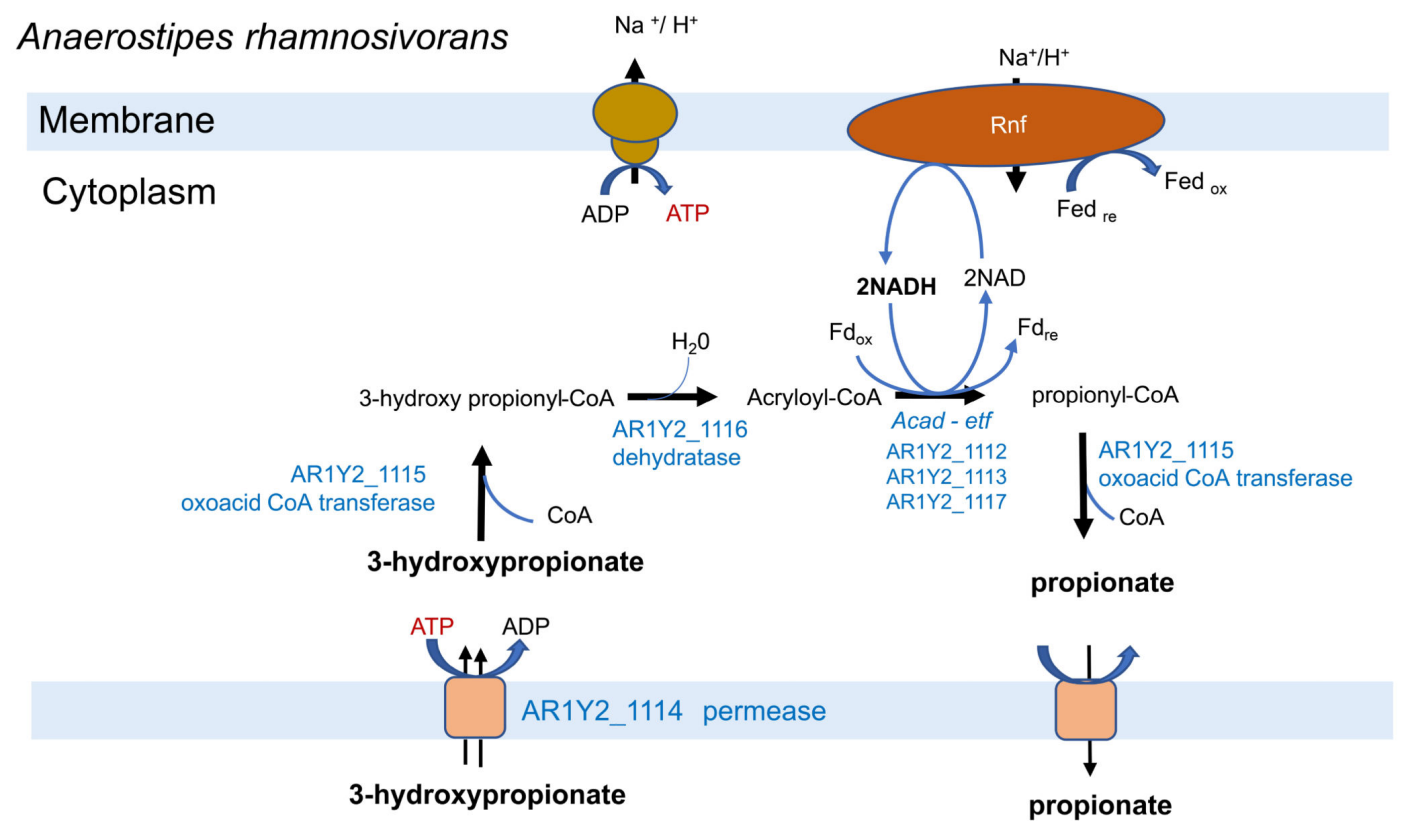
Reconstruction of 3-hydroxypropionate conversion to propionate by *A. rhamnosivorans*. 3-hydroxypropionate is imported *via* a permease (AR1Y2_1114) and further converted to 3-hydroxy propionyl-CoA, Acryloyl_CoA, propionyl-CoA and eventually propionate by an oxoacid CoA tranferase (AR1Y2_1115), a dehydratase (AR1Y2_1116), an acyl-CoA dehydrogenase/electron transfer flavoprotein complex (AR1Y2_1112, 1113 and 1117). This acyl-CoA dehydrogenase/Etf complex is predicted to be involved in energy conservation via by coupling the electrochemical gradient generated via a membrane bound Rnf complex with ATP synthesis.

**Extended Figure 7:**
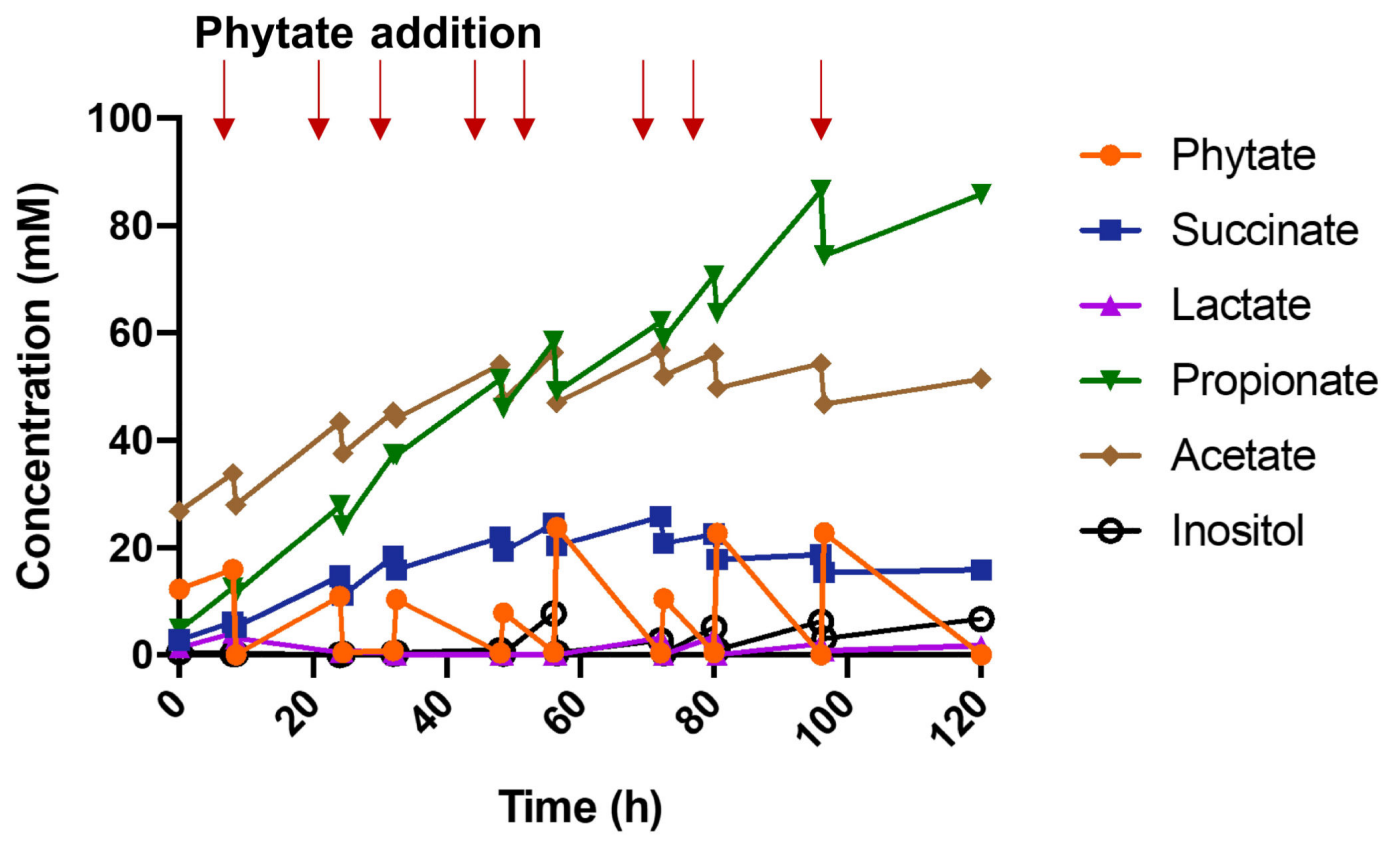
Production of bacterial supernatant of the co-culture for epithelial barrier function test using a Caco-2 cell model. The experiment was performed in YCFA medium supplemented with phytate. Around 20 mM phytate was repeatedly added after adjusting the pH to neutral (~7). Bacterial supernatant was collected before and after substrate addition for 5 days. In the end of the growth, propionate, acetate and succinate were found as major end metabolites with nearly 90 mM propionate. Red arrows indicate the time of phytate addition.

**Extended Figure 8:**
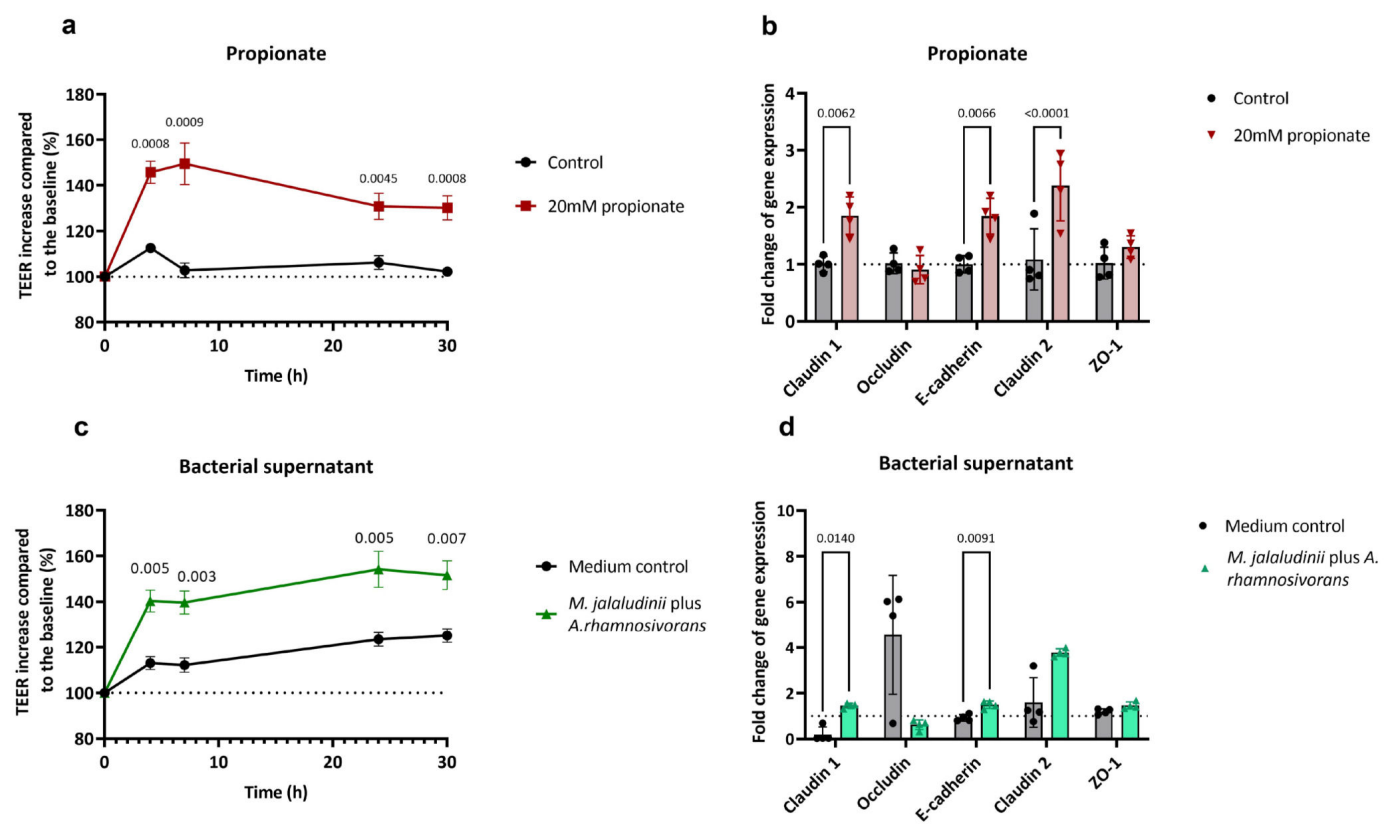
Propionate and phytate-derived bacterial metabolites improved epithelial barrier integrity via activating tight junction genes. **a:** 20mM propionate co-incubation increased TEER values as compared to the control. **b**: Increased activity of tight junction genes Claudin-1, Claudin-2 and E-cadherin upon propionate exposure. **c**: Increased barrier integrity when Caco-2 cells were incubated with 10 % bacterial supernatant from a coculture (*M. jalaludinii* and *A. rhamnosivorans*) compared to 10 % the medium control. **d**: Increased activity of tight junction genes Claudin-1 and E-cadherin upon bacterial supernatant exposure compared to the medium control. The fold change expression was normalized to the control used in c. Values in a and c are increases of TEER compared to the baseline (%). Data shown in b and d were normalized to the control of b. Data are presented as mean values +/- SD (n=4 biological replicates) and statistical analysis was performed by multiple Ttests (a,c) and two-way ANOVA (b, d). Only significantly different comparisons are shown with P values.

To further investigate the microbial interaction at the molecular level, we assessed the differential expression of phytate and *myo*-inositol degradation pathway genes of *M. jalaludinii* and *A. rhamnosivorans*, respectively, during growth in monocultures compared with co-cultures using transcriptomic analyses. The results of the metabolic measurements showed high concentrations of 3-hydroxypropionate only in monocultures of *M. jalaludinii* at the expense of high propionate concentration in the cocultures in both phytate and *myo*-inositol (Extended Fig. 5a-b). We found that the number of cells of the two bacteria were similar when grown on phytate while the cell numbers of *A. rhamnosivorans* were sixfold higher than those of *M. jalaludinii* with *myo*-inositol as a carbon source (Extended Fig. 5c-d). *A. rhamnosivorans* had weak effects on the overall transcription profiles of *M. jalaludinii* grown in phytate in contrast to strong influence on *M. jalaludinii* transcriptomic profile grown in *myo*-inositol (Extended Fig. 5e). For *M. jalaludinii*, the expression of genes involved in phytate dephosphorylation, inositol uptake, inositol fermentation was highly similar between monoculture and coculture in phytate (Fig. 4d, Table S3-4) while genes involved in *myo*-inositol uptake and utilization and 3-hydroxypropionate export (MJ_911) were up to 70-fold decreased in the coculture grown on *myo*-inositol, indicating reduced uptake and consumption of *myo*-inositol by *M. jalaludinii* due to the presence of *A. rhamnosivorans*. Comparing the whole transcriptome profiles of *A. rhamnosivorans* between cocultures and monocultures, we identified an entire operon (AR1Y2_1112-1117) involved in 3-hydroxypropionate transport (AR1Y2_1114) and conversion to propionate, which was increased up to 19-fold in coculture versus monoculture (Fig. 4e, Table S5-7). This observation confirms the active conversion of 3-hydroxypropionate to propionate by *A. rhamnosivorans* in coculture, which is in line with the metabolic observations. In contrast, the expression in *A. rhamnosivorans* of the inositol uptake gene (AR1Y2_0316) and the inositol utilizing operon (AR1Y2_1104-1107) was decreased around 15-fold in the cocultures, suggesting the reduced use of *myo*-inositol in the presence of *M. jalaludinii*. Based on these results, we reconstructed a model of metabolic interaction between *M. jalaludinii* and *A. rhamnosivorans* for phytate utilization (Fig. 4f and Extended Fig. 6). We subsequently analysed the functionality of the supernatant of the coculture of *M. jalaludinii* and *A. rhamnosivorans* on phytate and showed it to improve epithelial barrier integrity similar to propionate (Extended Figs. 7 and 8).

### In vivo phytate conversion by the microbial synergy

**Extended Figure 9:**
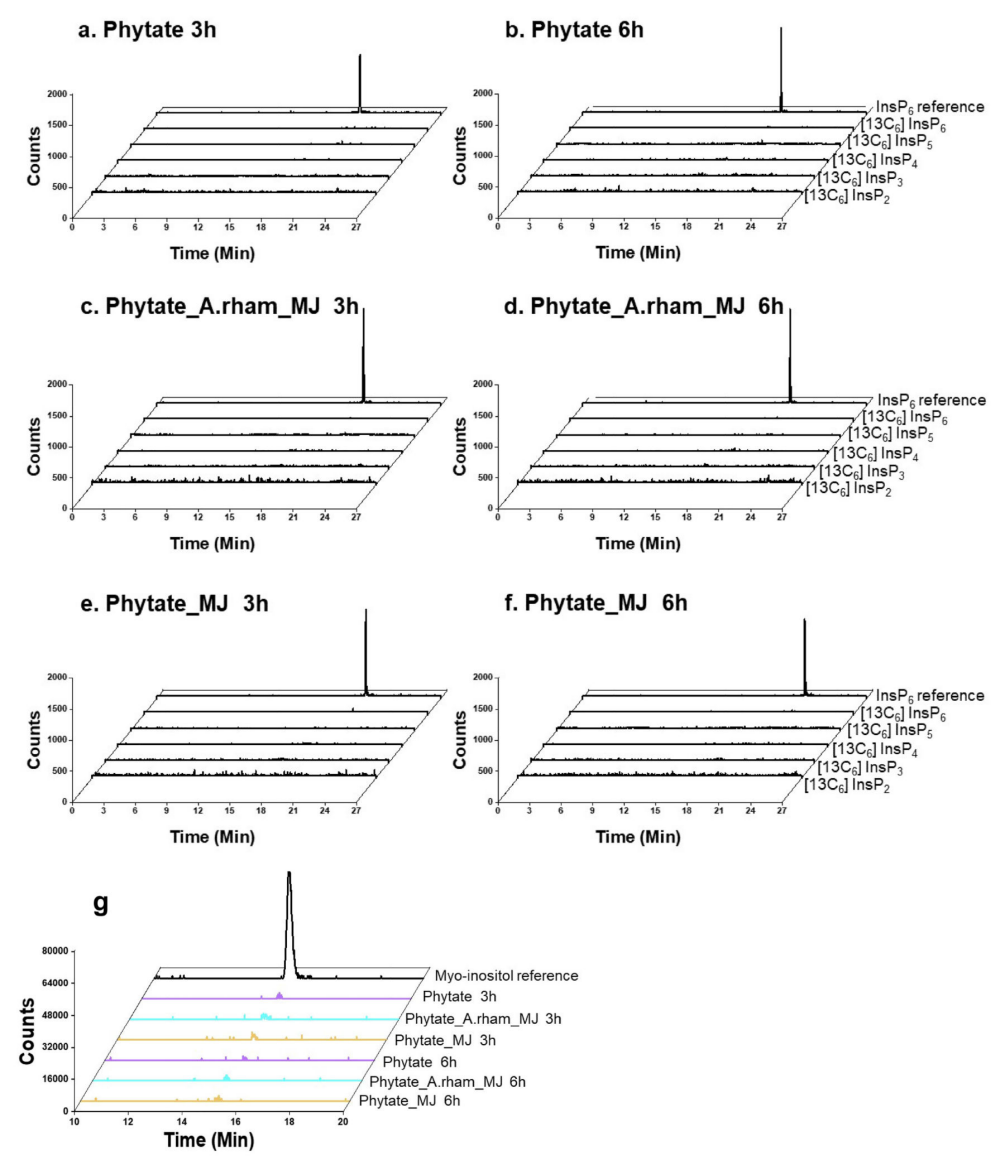
Targeted metabolomic analysis of [^13^C_6_]InsPs and [^13^C_6_]inositol in mouse plasma. Extracted counts of [^13^C_6_]InsP_2_, [^13^C_6_]InsP_3_, [^13^C_6_]InsP_4_, [^13^C_6_]InsP_5_ and [^13^C_6_]InsP_6_ in mouse plasma by CE-MS analysis are shown per treatment group. [^18^O_12_]InsP_6_ was used as reference in each measurement run. The limit of detection (LOD) was assessed via spiking with [^18^O_12_]InsP_6_ standard to TiO_2_ extracted plasma samples and calculation of a signal-to-noise above 3. The LOD value was 35nM in the extracted plasma matrix. Extracted Ion electropherograms depicting absence of individual [^13^C_6_]InsPs in a representative mouse of the phytate treatment group at 3 h (**a**) and 6 h (**b**); Phytate_A.rham_MJ treatment group at 3 h (**c**) and 6 h (**d**); phytate_MJ treatment group at 3 h (**e**) and 6 h (**f**). g, The measurement of [^13^C_6_]*myo*-inositol in the plasma samples by UHPLC-MS analysis indicated no [^13^C_6_]*myo*-inositol was detectable. [^12^C_6_]*myo*-inositol was used as reference in each measurement run. The LOD of *myo*-inositol was 8 nM as previously reported^66^

**Extended Figure 10:**
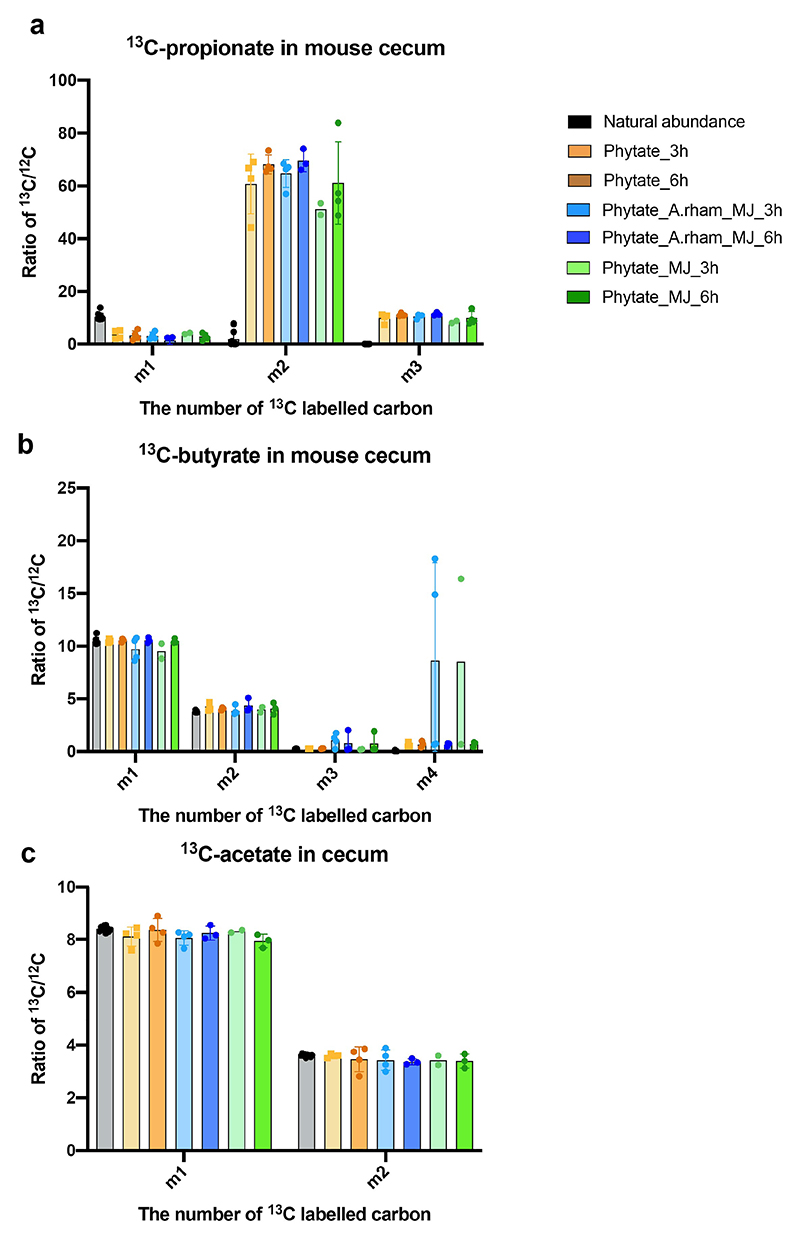
Enrichment of ^13^C-short chain fatty acids in mouse ceca upon bacterial oral challenge. Relative abundance of one ^13^C (m1); two ^13^C (m2); three ^13^C (m3) and four ^13^C (m4) of ^13^C-propionate (**a**); ^13^C-butyrate (**b**) and ^13^C-acetate (**c**) in mouse cecum in three treatment groups: Phytate (brown); Phytate_A.rham_MJ (blue) and phytate_MJ (green). Data are presented as mean values +/- SD (n=4 biological replicates for Phytate and Phytate_A.rham_MJ at 3h); (n=3 biological replicates for Phytate_A.rham_MJ and Phytate_MJ at 6h);(n= 11; 7; 10 biological replicates for natural abundances of ^13^C-propionate; ^13^C-butyrate and ^13^C-acetate respectively) or as mean values of two data points for Phytate_MJ at 3h due to technical issues during derivatization.

**Figure 5:**
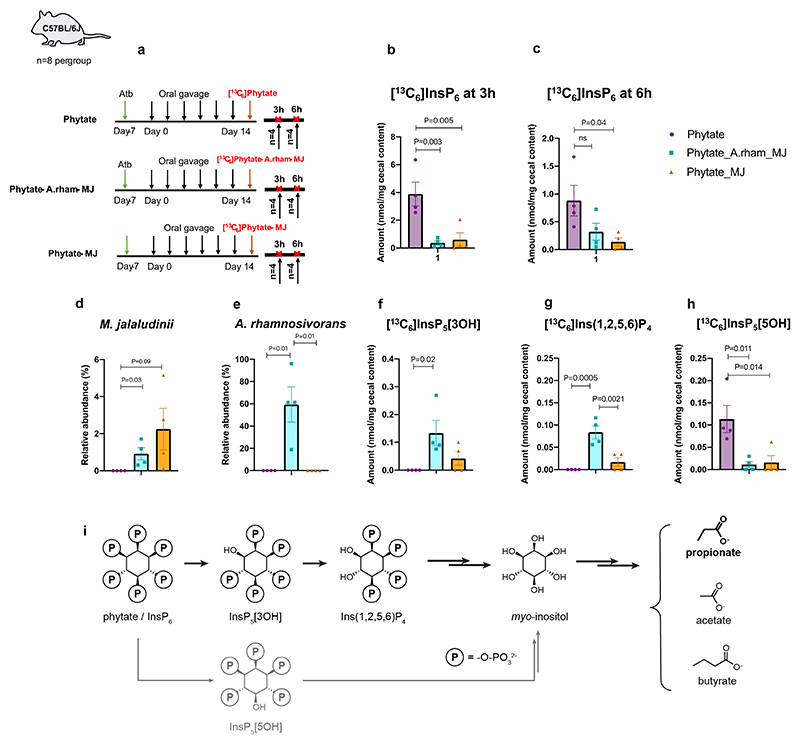
In vivo phytate conversion by *M. jalaludinii* and *A. rhamnosivorans* in mice. **a:** Experimental design of in vivo microbial phytate conversion. Three groups of 8 mice were treated with either phytate (0.1mg/g body weight) alone, or phytate (0.1mg/g body weight) together with *M. jalaludinii* (10^9 cells per dose) or phytate (0.1mg/g body weight) with *M. jalaludinii* (MJ) and *A. rhamnosivorans* (A.rham) (10^9 cells per strain per dose) every second day for two weeks. After two weeks, a [^13^C_6_]InsP_6_ oral challenge of the same bacterial dosage were performed for the respective group. A group of four mice was killed after 3 h or 6 h. Cecum and plasma samples were collected for inositol phosphate extraction and measurement while colon samples were used for quantification of *M. jalaludinii* and *A. rhamnosivorans* via qPCR. Significant lower levels of cecal [^13^C_6_]InsP_6_ in microbial treatment groups compared to control groups at 3 h (**b**) and 6 h (**c**) after oral challenge. **d**,**e**, Relative abundance of *Mitsuokella jalaludinii* at 6 h (**d**) and *A. rhamnosivorans* at 6 h (**e**) in murine colon samples quantified via qPCR in three groups: phytate (purple); phytate_A.rham_MJ (blue) and phytate_MJ (orange). **f-h**, Quantification of three [^13^C_6_]InsPs species: InsP_5_[3OH] at 6 h (**f**), Ins(1,2,5,6)P_4_ at 6 h (**g**); InsP_5_[5OH] at 3 h (**h**) in the cecum samples by BIRD-{^1^H,^13^C}HMQC spectra at 6 h and 3 h after oral challenge. Relative abundance of *A. rhamnosivorans* (**g**) and *Mitsuokella jalaludinii* (**h**) in colon samples quantified via qPCR in three groups: phytate (purple); phytate_A.rham_MJ (blue) and phytate_MJ (orange). **i**: Postulated phytate degradation routes in mouse cecum. Black arrows indicate the route involved *Mitsuokella jalaludinii* and *A. rhamnosivorans* while grey arrows are routes by the mouse microbiome alone. Two arrows indicate conversion required more than one reaction. Data are presented as mean values +/- SEM (n=4 biological replicates) and statistical analysis was performed using one-way ANOVA. Only significantly different comparisons are shown with P values.

We subsequently studied the in vivo synergy between *M. jalaludinii* and *A. rhamnosivorans* in mice that were gavaged with *M. jalaludinii* alone, both bacteria or a sterile control and challenged with [^13^C_6_]InsP_6_ (Fig. 5a). We observed that the cecal [^13^C_6_]InsP_6_ levels were significantly lower after 3 h and 6 h oral challenge in groups receiving bacteria (Fig. 5b-c), indicating the active conversion of phytate. Mice that received the bacteria had less than 0.5 nmol of [^13^C_6_]InsP_6_ per mg cecal content as opposed to 4 nmol of [^13^C_6_]InsP_6_ per mg cecal content from control mice after 3 h oral challenge. This was associated with high colonic levels of *M. jalaludinii* in bacterial treatment groups compared with the control group (Fig. 5d). Similarly, the colonic level of *A. rhamnosivorans* was high only the combination treatment (Fig. 5e). The difference of [^13^C_6_]InsP_6_ level between the treatment and control groups was smaller at 6 h after of oral challenge, suggesting the residual mouse microbiome was capable of degrading InsP_6_ but at a slower rate as compared to those with supplemented with *M. jalaludinii* (Fig. 5c). Importantly, no [^13^C_6_]InsPs and [^13^C_6_]-*myo*-inositol were detected in the mouse plasma at both 3 h and 6 h time points by CE-MS (Extended Fig. 9), indicating the microbiome was mainly responsible for dietary phytate consumption. Remarkably, we detected large enrichment of ^13^C-propionate but not ^13^C-acetate or minor ^13^C-butyrate in mouse cecal samples, indicating the major production of propionate from oral phytate (Extended Fig. 10). However, no significant difference in propionate accumulation was observed between the treatment groups and the control group, likely reflecting the its rapid absorption by colonocytes^43^ (Sup. Fig. 4a-b). The [^13^C]3-hydroxypropionate cecal level was significantly higher in the *M. jalaludinii* only but not the combination group compared to the control (Sup. Fig. 4c-d), indicating active conversion of phytate to 3-hydroxypropionate by *M. jalaludinii* and a moderate conversion of 3-hydroxypropionate to propionate by *A. rhamosivorans*. While [^13^C_6_]InsP_6_ was the most abundant inositol phosphate component in the mouse cecum (Sup. Fig. 5), smaller amounts (<0.25nmol/mg cecum) of other inositol phosphates (InsP_5_[3OH], Ins(1,2,5,6)P_4_ and/or its enantiomer Ins(2,3,4,5)P_4_ and InsP_5_[5OH]) were also detected in the cecum samples of the mice (Fig. 5f-h). Of note, InsP_5_[5OH] level was significantly lower in bacterial treatment groups (Fig. 5h) whereas InsP_4_ and InsP_5_[3OH] levels were higher in two treatment groups compared to the control group (Fig. 5f-g), implicating multiple phytate degradation pathways in presence and absence of these two administered bacteria. Based on these data, two main microbial phytate degradation routes in mouse cecum were deduced (summarized in Fig. 5i).

## Discussion

Phytate is abundantly present in plant-based diets, and notably abundant in nuts, seeds and unprocessed whole grain. These diets have shown to provide a panoply of health benefits in mice and human^10–11, 44–46^. However, the role of the human microbiome in phytate metabolism has not been well studied. It is largely presumed that dietary phytate is absorbed in the intestine or partially converted to highly dephosphorylated *myo*-inositol derivatives (InsP_3_; InsP_4_ and InsP_5_) via activities of the human microbiome^26–27^. Our previous finding highlighted the capacity of the fecal microbiome to convert phytate to different SCFAs without identification of the bacterial species involved in phytate degradation and how they interact with other bacteria for SCFA production^22^. Here, we showed that the human microbiome can convert phytate rapidly into different types of SCFAs depending on the microbial composition. In the current study, we identified *Mitsuokella* spp. as an efficient and prevalent phytate degrader in the human gut. This was demonstrated via in vitro and in vivo phytate consumption using a stable-isotope labeling approach (Fig. 3-5). This is in line with a previous report that *M. multacida* has an outer membrane located phytase^32^. We found that *Mitsuokella* is a prevalent genus in the general population cohort with *M. jalaludinii* as the most abundant species. *Mitsuokella* spp. were significantly correlated with the *Prevotella* enterotype, which has been associated with high fibre intake and good health^37–38^.

Using *M. jalaludinii* DSM13811^T^ as a model phytate-degrading species^40^ in conjunction with ^13^C-labelling of phytate, we demonstrated that *M. jalaludinii* was able to degrade dietary phytate in vitro and in vivo to produce a gut metabolite 3-hydroxypropionate^47^. It has indeed been reported that supernatant of *M. jalaludinii* cultures inhibited growth of *Salmonella* and reduced virulent factors but this study did not identify the responsible compound^48^. Our data indicate that the observed growth inhibition was caused by the production of 3-hydroxypropionate by *M. jalaludinii* (Fig. 3-4a) as 3-hydroxypropionate is known to exhibit strong anti-microbial properties^49^. The production of the toxic 3-hydroxypropionate may serve as a means to increase the competitiveness of *M. jalaludinii* in the colonic environment. This is supported by our observation that *A. rhamnosivorans* shows sensitivity to 3-hydroxypropionate but has developed a mechanism for its detoxification. By transcriptomic analysis we identified the induced genes involved in the entire phytate degradation pathway in *M. jalaludinii*, including a phytase, inositol utilization gene cluster, a succinate production gene cluster and an important 3-oxopropionate reductase for 3-hydroxypropionate production. The production of 3-hydroxypropionate by the human *M. jalaludinii* H1-1 strain also confirmed the active degradation of phytate by *Mitsuokella* spp. in faecal enrichment cultures where 3-hydroxypropionate was found as an intermediate (Fig. 1a and Sup. Fig. 6a, b)

We further demonstrated that *M. jalaludinii* formed a synergistic interaction with *A. rhamnosivorans* mainly via 3-hydroxypropionate (Fig. 4 and Sup. Fig. 6b, c), leading to the production of propionate from dietary phytate in vitro and in vivo. The metabolic pattern of phytate metabolism by these two bacteria mimicked the fecal phytate enrichment for which a rapid and consistent phytate degradation was observed over multiple transfers. This suggests this type of microbial synergy may be necessary for an efficient and persistent phytate degradation. Transcriptomic analyses indicated that *A. rhamnosivorans* had little influence on the activity of *M. jalaludinii* in the presence of phytate and mainly used released 3-hydroxypropionate via an identified scavenging pathway to produce propionate. Interestingly, we found that the bacterial supernatant of *M. jalaludinii* grown together with *A. rhamnosivorans* in phytate improved the barrier integrity via activation of tight junction genes similarly to treatment with propionate when being exposed to Caco-2 cells (Extended Fig. 8). Intestinal barrier disruption may lead to systemic infectious and inflammatory consequences of obesity and diabetes^50^. These results suggest that the production of propionate from phytate by *M. jalaludinii* and *A. rhamnosivorans* may provide metabolic benefits to the host. This is in line with our observation of the high prevalence of *M. jalaludinii* in the healthy subjects in the HELIUS cohort (Fig. 2) as opposed to the reduced abundance of *Mitsuokella* spp. in cardiometabolic participants in the LifeLines cohort^39^.

To detail the in vivo synergy between the two bacteria, we administered C57Bl/6J mice either phytate alone, phytate with *M. jalaludinii* or phytate with both *M. jalaludinii* and *A. rhamnosivorans* for two weeks and performed [^13^C_6_]InsP_6_ oral challenge to study the phytate metabolism at 3 h and 6 h after administration (Fig. 5). We found that [^13^C_6_]InsP_6_ and lower phosphorylated [^13^C_6_]InsPs were detected in the mouse cecum but absent in the plasma, indicating the microbiome was mainly responsible for dietary phytate metabolism. This is in line with previous reports that less than 20 % of phytate-derived inositol after oral phytate administration was detected in the mouse liver^21^. Three [^13^C_6_]InsPs were found in the mouse cecum after 3 h and 6 h oral challenge; InsP_5_[3OH] and InsP_4_ were enriched upon bacteria treatment while only InsP_5_[5OH] was enriched in [^13^C_6_]phytate-alone treatment. This suggests the *M. jalaludinii* not only significantly enhanced the phytate degradation capacity of the murine microbiome but also affected the intestinal phytate degradation pattern. Further studies, however, are needed to determine whether supplementation of *Mitsuokella* and *Anaerostipes* strains could improve phytate degradation and increase SCFA (mainly propionate) production, and the therapeutic doses to provide metabolic benefits in human.

In conclusion, we discovered a strong correlation between *Misuokella* spp. and phytate degradation in the human gut and showed the synergy of the prevalent *M. jalaludinii* with *A. rhamnosivorans* in phytate breakdown to SCFAs. This also suggests that the beneficial effects of dietary phytate on host health may derive from the modulation of the microbiome. Therefore, our work may promote strategic approaches employing this microbial synergy and dietary phytate to beneficially intervene in human human health.

### Online Methods

The research complies with all relevant ethical regulations; Academic Medical Center (AMC) Medical Ethics Committee and Amsterdam Medical Center approved the study protocols.

### Phytate degradation by fecal microbiome using stable isotope labeling

Fresh stools were collected from two healthy donors (one 59-year-old female and one 54-year-old male) of whom informed consents were obtained in the BARIA study; this work protocol was approved by the Academic Medical Center (AMC) Medical Ethics Committee. We have complied with all relevant ethical regulations for work with human participants. Fresh stool samples from donor A and B were collected in falcon tubes and quickly brought to an anaerobic chamber to prepare fecal suspension for inoculation. 0.2 ml of fecal suspension was added into 10ml bicarbonate buffered medium containing either 10 mM non-labelled phytate or [^13^C_6_]InsP_6_ as sole energy and carbon source. [^13^C_6_]InsP_6_ was synthesized and purified according to previously reports^33, 51^. Samples of [^13^C_6_]InsP_6_ enrichments were taken at several time points during a first few days after inoculation. The supernatants of these samples were used for ^13^C-NMR analysis to monitor substrate consumption and end metabolite production. [^13^C_6_]InsP_6_ was synthesized and purified according to previously reported^51^. The first non-labeled phytate enrichments were subsequently transferred to fresh media with none-labelled phytate as the sole substrate to further enrich for phytate-degrading microbes. At the third transfer, phytate degradation capacity and metabolite production were assessed by HPLC analysis on the bacterial supernatant over 5 days, while the pellets were collected for gDNA extraction followed by 16S rRNA sequencing analysis to determine changes of the microbial composition during phytate incubation. Stools of donors A and B were used to extract gDNA and inositol phosphates to further determine the microbial composition by 16S rRNA sequencing analysis and inositol phosphate profiles by 1D- and 2D-NMR analyses (see below for detailed protocol).

### 1D- and 2D-NMR measurements

All chemicals were used without further purification unless specified. Deuterated solutions (deuterium oxide (D_2_O, cat. D215T for lyophilized samples), sodium deuteroxide (NaOD, cat. D076Y) and deuterium chloride (DCl, cat. D070Z) were obtained from Eurisotop, deuterium oxide (D_2_O, cat. 00506-100mL, for spiking and non-lyophilized samples) from Deutero, perchloric acid (HClO_4_, cat. 1.00518.1001) from Supelco, potassium hydroxide (KOH, cat. 6751.1) from Carl Roth, ammonia solution (cat. 338818-100mL) from Sigma Aldrich, tetramethyl phosphonium bromide (TMPBr, cat. A17907) from Alfa Aesar, titanium oxide particles (Titanosphere 5 μm, cat. 5020-75000) from GL Sciences.

For NMR measurements and NMR data analysis TopSpin 3.5 was used. For formatting the processed spectra were loaded into MestreNova 10.0 and exported into Adobe Illustrator where lines tracing accumulation of metabolites in stacked spectra were manually added. Measurements were conducted on a Bruker AV-III spectrometer (Bruker Biospin, Rheinstetten, Germany) operating at 600 MHz for ^1^H and 151 MHz for ^13^C nuclei equipped with a cryo-QCI probe. The pulse sequence for BIRD-{^1^H,^13^C}HMQC is based on the hmqcbiph pulse program from Bruker. Measurement parameters are adapted depending on sample composition. Typically, HMQC measurements of lyophilized bacterial culture samples were recorded with TD(^13^C) = 512, 64 scans, spectral width (^13^C) limited to 50 – 90 ppm. HMQC measurements of cecal and plasma extracts were recorded with 128 scans, and P1 pulses determined for every single sample, and otherwise identical parameters. For the measurement of ^13^C-NMR spectra of bacterial culture samples TD = 131072, 2048 scans, and a spectral width of 220 ppm – -20 ppm were used. All samples were recorded at 310 K. BIRD-{^1^H,^13^C}HMQC-NMR spectra were processed without digital water suppression with manual phasing and automatic baseline correction. ^13^C-NMR spectra were processed with SI = 16384, manual phasing and segment-wise automatic baseline correction. Quantification of NMR data were conducted as follows: For cecal extracts InsPs were quantified against a known concentration of tetramethylphosphonium bromide (TMPBr). A standard curve for InsP_6_ against TMPBr was recorded earlier^51^. For other InsP species the standard curve for InsP_6_ was used as an approximation as there are no fully ^13^C-labeled standards available. As the signals of the 2-positions are the sharpest and best resolved (due to the reduced coupling to the neighbouring CH groups), the 2-position signals were used for quantification.

For the analysis of metabolites produced in bacterial cultures, capped NMR tubes were roughly sterilized by rubbing with 70 % ethanol and radiation with UV light for 20 min in an aseptic laminar flow hood. Thawed aliquots of bacterial culture samples were centrifuged (5 min, 5000 g, 4 °C) and 500 μL of the supernatant were transferred under aseptic conditions into a sterilized NMR tube and 55 μL of D_2_O (from a previously unopened bottle which was kept under aseptic conditions) were added for locking during NMR measurements. Samples were kept at 4 °C until the measurement of ^13^C-NMR spectra on the same day. NMR samples which contained multiple InsP species according to the ^13^C-NMR were lyophilized, redissolved in D_2_O, and lyophilized again. Finally, the samples are taken up in 500 μL of D_2_O and pH adjusted to 6.0 with NaOD and DCl and submitted for recording HMQC and HMQC-CLIP-COSY spectra.

### Microbiome profiling analysis

DNA from using from 1ml bacterial culture of fecal phytate enrichments using a repeated bead-beating protocol^52^. 16S rRNA amplicon libraries were generated with a single-step PCR targeting the V3-V4 region^53^. Amplicons were sequenced using an Illumina MiSeq with V3 chemistry and 2x251 cycles. For the enrichments 16S V3-V4 amplicon sequences were parsed using a vsearch^54^ (v2.15.2) based pipeline. Paired end reads were merged, with max differences set to 100 and allowing for staggered overlap. ASVs were inferred from reads with lower than 1.5 expected error rate using the ‘cluster unoise with centroids’ algorithm with a minsize of 4, after which chimeras were removed using the uchime3 denovo method. For each sample ASV abundances were determined by mapping the merged reads against the ASV sequences with identical matches sequence set using the usearch_global algorithm with a 0.97 distance cut off. Taxonomy was assigned using R (V4.0.5) and the dada2^55^ assign taxonomy function using the silva (v132)^56^ reference database (arb-silva.de).

### Human cohort study

The HELIUS study is a population-based multi-ethnic prospective cohort study, based in Amsterdam, The Netherlands. Baseline data collection took place between 2011 and 2015 among the six largest ethnic groups in Amsterdam (those of Turkish, Moroccan, African Surinamese, South-Asian Surinamese Ghanaian and Dutch origin). People in these groups who were aged 18-70 were randomly, stratified by ethnicity, recruited from the municipal registry. Data collection consisted of a questionnaire and a physical examination including the collection of biological samples. Faecal samples were collected from participants. Microbiome data from the HELIUS study was processed as previously described^57^. ASV variants with an average abundance of more than 0.1 % were selected as representatives. Sequences were placed phylogenetically placed in the tree from the Living tree project (LTP_06_2022) using MAFFT (V7.310) and Fastree (V2.1.11). The tree was subset to all members of the *Selenomonadales* order. Ordination of the microbiome was performed by principal coordinate analysis of the Bray-Curtis dissimilarity. Enterotypes were defined by pam-clustering of the Jensen-Shannon Distance and were labelled by their major representative clade. Association of the ASVs with the different principal coordinates was determined by calculating the weighted average scores using wascores from the vegan (V2.6.4) package. Differences in prevalence were tested with a chi-square test. Data were visualized using ggplot2. The HELIUS study complies with all relevant ethical regulations, is in accordance with the Declaration of Helsinki (6^th^, 7^th^ revisions), and the study protocol is approved by the Academic Medical Center Medical Ethics Committee. All participants provided written informed consents. The participants were voluntarily enrolled in this study without compensation.

### Isolation of prevalent *Mitsuokella* strain

The stool sample of donor A was suspended in anaerobic PBS buffer (P2272, Sigma Aldrich) in an anaerobic tent to make a serial dilution of fecal slurry from 10^-1^ to 10^-5^. A volume of 200 μl of these dilutions were smeared on a screening agar medium containing sodium phytate (P8810, Sigma Aldrich) as the sole energy and carbon source. The screening medium was prepared by saturating 20mM phytate with magnesium chloride (7786-30-3, Sigma Aldrich) to produce milky agar plates. The agar plates were subsequently incubated in an anaerobic jar filled with N_2_/CO_2_ at 37 °C up to 5 days. Colonies surrounded with clear zones as indication of phytate degradation activities were picked up and streaked on new agar medium to confirm the degradation activity. This streaking was repeated a several times before being inoculated in a liquid medium containing phytate as the sole energy and carbon source. The identity of the isolate was determined via 16S rRNA sequencing at Macrogen and a draft genome of the *Mitsuokella jalaludinii* isolate was sequenced by Illumina NovaSeq 6000 S4 PE150 XP and assembled using Genome de novo assembly pipeline at Eurofins. The genome of *Mitsuokella jalaludinii* H1-1 isolate is publicly available on the NCBI genome database (BioProject: PRJNA1032471)

### Phylogeny of phytases

A phytase phylogenetic tree was constructed from amino acid sequences of phytases and phosphatases from all hosts ranging from mammals to bacterial species that were retrieved from the NCBI database. This included previously reported phytase and phosphatase amino acid sequences from all hosts^58^, *E. coli* phytase^9^, *Akkermansia* phytase^59^ and *Mitsuokella* phytase^32^. All amino acid sequences were aligned using the Clustal_X programme. A phylogenetic tree was constructed using the neighbour-joining algorithm by the MEGA 7 with 1000 bootstraps to obtain confidence levels for the branches.

### Bacterial culturing

*Mitsuokella jalaludinii* DSM13811^T^ was obtained from DSMZ culture collection and *Anaerostipes rhamnosivorans* 1y-2^T^ was isolated previously and is available as DSM26241^T^. These two bacteria were routinely maintained in a modified YCFA medium^22^ supplemented with *myo*-inositol (87-89-8, Sigma Aldrich) for A. *rhamnosivorans* or sodium phytate (P8810, Sigma Aldrich) for *M. jalaludinii*.

The growth experiments were performed in duplicate in 20 ml bicarbonate-buffered medium^60^ supplemented with 20 mM phytate filled with N_2_/CO_2_ (80:20, v/v) gas in the head phase. Phytate and inositol were filter sterilized as stock solutions of 0.2 M and 0.5 M. respectively. The condition in which the bacteria were added to 20 ml bicarbonate-buffered medium without substrate was used as control. The growth was monitored via metabolite formation by HPLC and optical density measurement by a spectrophotometer at a wavelength of 600 nm.

### Monoculture and coculture studies of phytate degradation

The coculture study was performed in a bicarbonate buffered medium supplemented with phytate as sole energy and carbon source. Monoculture of *M. jalaludinii* or coculture of *M. jalaludinii* and *A. rhamnosivorans* were inoculated with 10 mM phytate. Bacterial supernatants were collected from both conditions at several time points for substrate consumption and metabolite production by HPLC. To identify the intermediates of phytate degradation by the monoculture and the coculture, the bacteria were inoculated with 5 mM [^13^C_6_]InsP_6_ and bacterial supernatants were collected during the growth for NMR measurements. *M. jalaludinii* DSM13811^T^ and *A. rhamnosivorans* 1y-2^T^ were pre-cultured in YCFA medium supplemented with 20 mM phytate or 20 mM *myo*-inositol respectively. These precultures were added with 2.5 % to the media of the test conditions.

To investigate the synergy between *M. jalaludinii* and *A. rhamnosivorans*, the bacteria were grown in mono culture and coculture in a medium containing 10 mM, 20 mM and 40 mM phytate or 10 mM, 20 mM or 40 mM *myo*-inositol in 96-well plate incubated in an anaerobic tent for growth monitoring for 48 h. At the end of the growth period, the bacterial supernatants were collected to analyze the substrate consumption and metabolite production. The experiment was performed in biological triplicates.

To study the growth rate of *M. jalaludinii* in phytate and *myo*-inositol; the bacteria were inoculated in YCFA medium supplemented with either 10 mM or 40 mM phytate or 10 mM or 40 mM *myo*-inositol. The experiment was performed in 96-well plate which was incubated at 37 °C in a plate reader in the anaerobic chamber to monitor the OD_600_ every 30 min for 24 h. The doubling time was determined from OD_600_ readings during exponential growth.

To study the capacity of *A. rhamnosivorans* to use 3-hydroxypropionate, the bacterium was incubated in a bicarbonate buffered medium containing 10 mM 3-hydroxypropionate or 15 mM glucose or both. The cultures were collected during 48 h for OD_600_ and end metabolite measurements. The experiment was performed in biological duplicate. To study the influence of 3-hydroxypropionate on the growth on *myo*-inositol, *A. rhamnosivorans* was grown in 20mM *myo*-inositol supplemented with either 0, 10, 20, 30, 40, 50 mM 3-hydroxypropionate and the growth was monitored by OD_600_ measurement every 30 minutes in a plate reader placed in an anaerobic tent. The experiment was performed in biological triplicates.

### Analytical methods

Phytate, *myo*-inositol, succinate, 3-hydroxypropionate and other short-chain fatty acids and alcohols were quantified on a Shimadzu HPLC system equipped with a Shodex sugar SH1821 6 μm, 8.0 x 300 mm column. The column was kept at 45 °C while running with 0.005 M H_2_SO_4_ as eluent under a flow of 1 ml/min. The detector was a refractive index detector. Chemicals at HPLC quality were used to prepare the standard curves. All analyses were performed in duplicate.

### Transcriptomics

To identify genes involved in phytate degradation by *M. jalaludinii* DSM13811^T^, the bacterium was grown in two conditions including a bicarbonate buffered medium supplemented with 20 mM phytate or 20 mM *myo*-inositol. The experiments were performed in triplicate. The bacteria were harvested after 17 h incubation. Bacterial supernatants were collected to perform HPLC measurements of substrate consumption and metabolite production while the cell pellets were used for RNA extraction. Cells were harvested from 100 ml bacterial cultures by centrifugation at 4700 g for 30 min at 4 °C in 50 ml-pre-cooled sterile falcon tubes. Pellets were washed with 20 ml, 20 mM TE-buffer (pH 7) at 4700 g for 30 min at 4 °C and re-suspended in 150 μl TE buffer. Cell suspension was incubated with Lysozyme at 37 °C for 10 min. Cell lysis and RNase inactivation was performed by addition of a mix containing 4 μl β-mercaptoethanol, 1 μl proteinase-K and 150 μl of Gram-positive lysis solution (Gram positive DNA extraction kit, Masterpure). Lysis was done at 65 °C for 15 min while vortexing every 5 min. After incubation, the mix was quickly cooled on ice for 5 min and proteins were precipitated by adding 175 μL of MPC protein precipitation reagent (Gram positive DNA extraction kit, Masterpure). Debris was removed via centrifugation at 4 °C 10000 g. The sample was further cleaned and purified via the automated Maxwell LEV simply RNA extraction kit (Promega, Madison, USA), according to manufacturer’s instructions. Quality and quantity of the RNA extracts was checked by spectrophotometer DS-11 FX (DeNovix) according to manufacturer’s instructions. RNA was collected in RNase free water and stored at −80 °C till further analysis. Depletion of rRNA and RNA quality were again assessed by a Nanodrop (A260/280 ~ 2.0 and A260/230 ~ 2.0-2.2) and a fragment analyzer (Agent 5400) at Novogene (Hong Kong, China). The RNA samples once passed the QC for integrity and quality check, were subjected for whole transcriptome sequencing. Paired-end 150bp libraries were generated by Illumina NovaSeq 6000 sequencing system.

To investigate the interaction between *M. jalaludinii* DSM13811^T^ and *A. rhamnosivorans* in phytate at molecular levels, *M. jalaludinii* and *A. rhamnosivorans* were grown either together or alone in a bicarbonate buffer medium supplemented with 20 mM phytate or 20 mM inositol. The experiments were performed in triplicate. The bacteria were harvested after 17 h incubation. Bacterial supernatants (1ml) were collected to perform HPLC measurement for substrate consumption and metabolite production, while the cell pellets were used for quantification of *M. jalaludinii* and *A. rhamnosivorans*. qPCR primers for *M. jalaludinii* were designed to quantify *M. jalaludinii* in the cocultures and murine microbiome using Primer3 and Blast based on 16S rRNA sequence of *M. jalaludinii* DSM13811^T^. The primers were validated using gDNA of *M. jalaludinii*. Primers for *M. jalaludinii* were MJ_F1: 5’-TCTGTTGTCGGGGACGAATG-3’ and MJ_R1:5’- ACGTAGTTAGCCGTGGCTTC-3’, which resulted in a 83 bp amplicon. The 16S rRNA gene of *M. jalaludinii* was used to optimize temperature and make standard curves. The qPCR programme was 95 °C for 5 min and 35 cycles consisting of 95 °C for 30 s, 60 °C for 10 s and 72 °C for 40 s; 95 °C for 1 min and 60 °C for 1 min. DNA copies were calculated based on standard curves. qPCR primers for *A. rhamnosivorans* were used as previously designed and validated^22^. Bacterial cultures (100 ml) were used for RNA extraction and sequencing as described above. Obtained reads were aligned against reference genomes (BioProject PRJNA223472) of *M. jalaludinii* and *A. rhamnosivorans* using bowtie2 (V2.5.1). Gene counts were gathered in R (V4.0.5) using feature counts function of the R subread package (V4.3). Differential expression analysis was performed using DESeq2 (V 1.38.3). For analysis were re-annotated using an available genome from GenBank (BioProject PRJNA223472) and re-annotating the genome in Rapid Annotation using Subsystem Technology (RAST) (Table S3).

To select the proposed pathway genes, we first looked at genes with high homology to known genes that have been biochemically characterized. For examples, inositol utilization genes were identified via proteome analysis^22^ and biochemical analyses^61^. The function of key proteins was also verified by BLASTing the amino acid sequences in Pfam, InterPro, Brenda and Uniprot databases. Subsequently, we confirmed that these genes were detected in the transcriptome at highest gene counts, indicative of high expression in a given condition on inositol and phytate. Genes chosen were highly expressed in both condition (phytate and myo-inositol). For example, a few gene copies of inosose dehydratase were found in the genome, two of which (MJ_0910; MJ_0385) were detected in the transcriptome, and the expression of MJ_0910 was detected with around 13000 gene counts while only 48 counts were assigned for gene with a locus tag MJ_0385. This indicated that MJ_0910 was actively involved in inositol utilization. In this case, we observed that the entire inositol utilization operon (MJ_0906-0910) were highly expressed in both conditions with high gene counts, indicating their active involvement in phytate and inositol metabolism. This process was repeatedly done to identify all genes of the proposed pathway.

### Animal studies using a stable isotope approach

All animal experiments were done in 4-week-old male C57Bl/6J mice (Charles River) housed in individually ventilated cages with 2 mice per cage and 24 mice for all treatments in total. Sample size was calculated using an online tool (http://www.lasec.cuhk.edu.hk/sample-size-calculation.html). With a power of 0.8 and alpha of 0.5, 4 mice per treatment group were needed to obtain statistical significance. This sample size was in line with the previous publication on significant difference of intestinal phytate degradation^9^. Mice were kept under constant temperature (19–21 °C); humidity of 40–70 % and a 12-h light/dark cycle with free access to food and water. The mice were on high-fat diet (D12492i, Bio Services) during the treatment. Food and water were renewed once a week. An enrichment with house and tissues were also provided for the mice. All animal experiments were conducted according to guidelines of the ‘Guide to the Care and Use of Experimental Animals’ approved by the Ethics Committee on Animal Care and Use in Academisch Medisch Centrum, the Netherlands. We complied with all relevant ethics regulations for animal testing and research.

A stable-isotope approach was employed to study in vivo conversion of phytate by *Mitsuokella jalaludinii* (MJ) and *Anaerostipes rhamnosivorans* (AR). Mice were received at the age of 4 weeks and randomized and divided in 2 mice per cage. Acclimatization time was 2 weeks. To reduce the interference of endogenous murine microbiome, mice were treated with antibiotic cocktail of 1 mg ml^−1^ ampicillin, 5 mg ml^−1^ streptomycin and 1 mg ml^−1^ colistin via oral gavage one time in 1 week prior to the intervention as described previously^9^. To test the in vivo degradation of phytate MJ and AR, 0.2 ml oral administration of 0.1 mg/g body weight phytate or 0.1 mg/g body weight phytate and 10^9^ c.f.u.s of MJ or 0.1 mg/g body weight phytate and 10^9^ c.f.u.s of MJ and 10^9^ c.f.u.s of AR were given to a group of 8 mice every second day for 2 weeks. Bacterial suspension was prepared in 10 % trehalose as described previously^62^. In week 3, [^13^C_6_]phytate-AR-MJ, [^13^C_6_]phytate-MJ or [^13^C_6_]phytate challenge were given to a group of 8 mice in corresponding group. To estimate the time preference of in vivo conversion of [^13^C_6_]InsP_6_, a vial of bacterial suspension of MJ and AR was inoculated in a medium supplemented with 5 mM phytate. Phytate conversion by the bacteria was monitored during 24 h via metabolite measurement by HPLC. The experiment was performed in biological duplicate. Each 4 mice per group were sacrificed after 3 h or 6 h of the ^13^C challenge, and cecum contents and blood were collected for targeted metabolomics analyses via NMR, CE-MS, GC-MS and UHPLC-MS/MS measurements. Detailed methods were described in following sections. Colon samples were collected for quantification of *A. rhamnosivorans* and *M. jalaludinii* by qPCR at 3 h and 6 h time points as described above. The oral gavage and data collection were randomized every week as well as in ^13^C challenge test in the end of the study. The data met the assumptions of the statistical tests used, including normality and equal variances were formally tested. Data collection and analysis were performed blind to the conditions of the experiments. All animals and data points were included in the analyses. All figures were made using Prism 8 (Graphpad).

### Extraction of InsPs from mouse cecal and plasma samples for metabolomics

The protocol for the extraction of InsPs from cecal samples was adapted from published procedures but scaled to match the higher mass and volume (between 20-120 mg)^63–64^. The cecum content is resuspended in ice-cold 1 M aqueous perchloric acid (HClO_4_, Supelco, 0.1 mL per 1 mg cecum content) in a 5 mL or 15 mL tube by pipetting up and down (using a 1 mL pipette tip with the tip slightly cut off to widen the opening) and vortexing for ~20 s until all major clumps have broken up. The suspension was then incubated for 20 min at 4 °C on a rotary shaker. The sample is then centrifuged (10 min, 21 000 g, 4 °C) in 2 mL tubes and the supernatant transferred into a separate 5 mL or 15 mL tube containing TiO_2_ beads (Titanosphere 5 μm, GL Sciences, 0.5 mg of TiO2 per 1 mg original cecum content material), which were already washed with 1 mL Milli-Q^®^ water and 1 mL 1 M perchloric acid. The extract and TiO_2_ beads were mixed by briefly vortexing and on a rotary shaker for 20 min at 4 °C. After centrifugation (10 min, 18 000 g, 4 °C) in fresh 2 mL tubes the supernatant was discarded (note: for transferring the supernatant without disturbing the TiO_2_ beads a 2 μL Eppendorf tip attached to the tip of a 1 mL tip was used with slow pipetting) and the beads united in a single 1.5 mL tube by resuspending in 1 mL HClO_4_ in total. After vortexing the suspension was again centrifuged (10 min, 18 000 g, 4 °C) and the supernatant discarded. To eluate InsPs from the TiO_2_ beads, the beads were incubated with 375 μL of 10 % ammonia solution for 5 min at room temperature on a rotary shaker. After centrifugation (IKA miniG, 2000 g, 1 min) the supernatant was collected in a separate tube. The elution step is repeated once more and the eluates are combined. The combined eluates are filtered through a 0.2 μm syringe filter (Sartorius Minisart RC4, cat. 17821-Q) which was subsequently rinsed with 150 μL of Milli-Q® water. The filtrate was collected in a new 1.5 mL tube and lyophilized. To reduce the water content for NMR analysis the lyophilized eluates were redissolved in 500 μL D_2_O and lyophilized again. The lyophilized samples were taken up in 500 μL D_2_O and defined volumes of NaOD and DCl were used to adjust the pH to 7.0. Afterwards, 2.86 μL of a TMPBr stock solution (20 mM in D_2_O) were added for quantification and submitted for NMR measurement.

The extractions of InsPs from plasma samples were performed based on the procedures of TiO_2_ purification method as reported^65^. Briefly, 60-100 μL of plasma was thawed on ice and an equal amount of perchloric aid (2 M, 4 °C) was added. The sample was rotated for 30 min at 4 °C and then centrifuged (15.000 g, 10 min, 4 °C). Transfer the supernatant to pre-washed TiO_2_ beads (5 mg per sample, 5020-75000 GL Sciences) and incubate at 4 °C with rotation for 20 minutes. After that, the suspension was centrifuged (3.500 g, 1 min, 4 °C) and the supernatant was discarded. Wash the unbinding compounds with 0.5 mL perchloric acid (1 M) twice. For elution, NH_4_OH (200 μL, 3 % [v/v]) was used. The elution step was repeated and the eluents were combined. The combined eluents were centrifuged (17.000 g, 1 min, 4 °C) to remove any insoluble residues. For subsequent CE-ESI-MS analysis, the supernatant was completely dried under vacuum evaporation (60 °C) and dissolved into 20 μL of ultrapure water before measurement.

### Extraction of *myo*-inositol from plasma samples for metabolomics

The extractions of inositol from plasma samples were performed based on the procedures as previously reported^65^. Briefly, plasma samples were thawed and vortexed before transferring 50 μL to 250 μL of acetonitrile containing 1 % of formic acid for protein precipitation. After centrifuging, the supernatants were transferred into a HPLC vial for the UHPLC-MS analysis.

### Capillary electrophoresis combined with triple quadrupole with electrospray ionization source analysis of [^13^C_6_] InsPs in plasma samples

Known amounts of isotopic standards (2 μM [^18^O_2_] 5-InsP_7_) were spiked into samples for quantitation of [^13^C_6_] PP-InsPs and [^18^O_12_] InsP_6_ (2 μM) were spiked into samples for quantitation of [^13^C_6_] InsPs. For plasma samples, 2 μM [^18^O_2_] 5-InsP_7_ and 2 μM [^18^O_12_] InsP_6_ were spiked. For cecum samples, the samples were diluted 20-fold, then 4 μM [^18^O_2_] 5-InsP_7_ and 20 μM [^18^O_12_] InsP_6_ were spiked. A CE-ESI-QQQ system is used for the measurement^15, 66^. Set MS source parameters and MRM transitions is provided in supplementary information. The injection for each sample is 30 nL.

### ^13^C-*Myo*-Inositol measurement in plasma samples

Plasma [^13^C_6_]*myo* inositol was quantified using UHPLC-MS/MS. Analysis was performed on a UHPLC (Agilent 1290 infinity II) coupled to a Q-TOF (Agilent 6546). Chromatographic separation was achieved using gradient elution on an UHPLC Acquity BEH Amide column (1.7 μm, 2.1 mm x 150 mm) at 30 °C at a flow rate of 0.4 ml/min. Mobile phase consisted of 0.04 % NH4OH (mobile phase A) and 100 % acetonitrile (mobile phase B). Setting of instrument parameters and gradient elution as shown in table. The elution started with 13 % mobile phase A, increased to 17 % mobile phase A between 0 and 16 min then to 19 % mobile phase A between 16 to 19 min. Between 19 to 21 min, mobile phase A was increased to 30 % then held for 2 min. Stock solution of *myo*-inositol (2 mg/mL) was prepared in ultrapure water, then was diluted in 87 % acetonitrile (starting mobile phase condition for analysis) to obtain five calibration levels (0.1 μg/mL, 0.5 μg/mL, 1 μg/mL, 5 μg/mL, 10 μg/mL) for quantification of myo-inositol in plasma. Each calibration levels have three replicates.

### Cecum SCFA measurement

SCFA concentration and ^13^C-enrichment in cecal sample were analyzed as described previously^67^. Briefly, samples were thawed, diluted in 500 μL PBS and spiked with 50 μL of (0.5 mg/mL) 2-ethyl butyric acid as internal standard. After adding 10 μL of HCl (12M), samples were homogenized and centrifuged (20 min, 15 000 x *g*, 4 °C). The supernatant was transferred to a glass vial with a spatula tip of solid NaCl. SCFAs were extracted in 2 mL diethylether. For SCFA derivatization, 500 μL of diethyl ether was derivatized with 50 uL N-tert-butyldimethylsilyl-N-methyltrifluoroacetamide (MTBSTFA) at room temperature overnight. For 3-hydroxypropionate, 1 ml of the diethyl ether was derivatized with N,Obis(trimethylsilyl)trifluoroacetamide (BSTFA) at 50 °C for 1 hour. Mass spectrometry analysis was performed using an Agilent 5975C series gas chromatography/mass spectrometry (GC-MS) (Agilent Technologies, Santa Clara, USA) equipped with a ZB-1 column (Phenomenex, Torrance, USA). The mass isotopologue spectra of ([M-57]^+^) fragment of the derivatives of acetate (m/z 117-1119, m_0_-m_2_), propionate (m/z 131-134, m_0_-m_3_), butyrate (m/z 173-149, m_0_-m_4_) and 3-hydroxypropionte (m/z 219-222, m_0_-m_3_) were monitored. The standard curves were prepared in a range of 10 μM to 10 mM for acetate, 3 μM to 3 mM for propionate and butyrate; 0.5 μM to 30 μM for 3-hydroxypropionate. These ranges covered all measured points.

### Caco-2 cell testing

To investigate the effects of microbial metabolites on barrier integrity, TEER (Transepithelial electrical resistance) experiments in Caco-2 cells were performed. Caco-2 cells were ordered from ATCC under accession number HTB-37. As propionate is the major metabolite from phytate break-down by the coculture, effect of propionate on barrier integrity was assessed in Caco-2 cell with transwell setting. To produce bacterial supernatants for Caco-2 cell work, a coculture of *M. jalaludinii* and *A. rhamnosivorans* was grown in YCFA containing 20mM phytate. The effects of propionate and bacterial metabolites on barrier integrity is expressed by TEER increase compared to the baseline (%). The pH of the cultures was monitored every day and adjusted to neutral pH after addition of substrates. The pH adjustment and substrate addition were conducted every day for 5 days. Bacterial supernatants were collected at all time points for HPLC measurement.

At 80 % of confluence of the Caco-2 cell culture, cells were treated with Trypsin-EDTA and seeded apically at 5x10^4^ cells per well onto 12 mm ThinCert™ with 0.4 μm pore membrane (Coming, CLS3460) in a 24-well plate. Growth media were renewed every 2 days with DMEM containing 10 % fetal bovine serum (FBS), 1 % penicillin-streptomycin and 1 % of non-essential amino acids. Caco-2 cells were incubated in an incubator maintained in a humidified atmosphere at 37 °C with 5 % CO_2_. Caco-2 cells were seeded on transwells for 21 days to obtain a monolayer for TEER measurement. TEER analyses were performed at day 7, 14, and 21 in culture. After 21 days, the medium in the apical compartment was replaced with medium containing either 20mM propionate; 10 % *M. jalaludinii* and *A. rhamnosivorans* supernatant or 10 % YCFA medium as control. TEER measurement was performed at T_0_; 4 h; 7 h; 24 h and 30 h. Cells with fresh medium was used as control condition. At the end of the experiment, the supernatant in the apical compartment was collected for HPLC analysis while Caco-2 cells were washed with 300 uL of PBS and collected in 300 uL of Trizol/Tripure®. These cells were then stored at -80 °C freezer for RNA extraction and qPCR to quantify the activity of tight junction genes. The experiment was performed in biological triplicate.

In order to assess the influence of propionate and bacterial metabolites on barrier integrity, total RNA was extracted from cells incubated with either water or 20 mM propionate, 10 % bacterial supernatant of the coculture of *M. jalaludinii* and *A. rhamnosivorans*, 10 % bacterial medium using TriPure reagent according to manufacturer’s instruction. Quality and concentration of extracted RNA were determined by a Nanodrop spectrometer. The cDNA was amplified using SensiFAST SYBR Green PCR Kit (Meridian bioscience, USA), following the manufacturer’s instructions. Human tight junction genes (Claudin 1, Occludin, E-cadherin, Claudin 2 and ZO1)^68^ were amplified using specific primers, with expression being normalized to 18S and 36B4 genes. The sequences of the primers are provided in the supplementary information. The qPCR programme was 10 minutes at 95 °C, 39 cycles consisting of 15 seconds at 95 °C and 30 seconds at 60 °C and melting curves were obtained at between 65 °C to 95 °C with an increment of 0.5 °C every 5 seconds. Fold changes were calculated using 2^-ΔCq^. Cq is the number of amplification cycles that is needed to reach a threshold of fluorescence signal. qPCR was performed with technical duplicates.

## Extended Data

## Supplementary Material

Supplementary information

## Data Availability

Annotated genome of *Mitsuokella jalaludinii* DSM13811T (BioProject PRJNA223472) as Table S3. A draft genome of *Mitsuokella jalaludinii* H1-1 isolate has been deposited in NCBI genome database (BioProject: PRJNA1032471). Taxonomic assignment of the phytate microbial communities was performed using the silva (v132) as reference database (arb-silva.de). The phylogenetic tree of *Mitsuokella* ASVs was based on the living tree project (https://imedea.uib-csic.es/mmg/ltp/#Downloads). The raw reads from transcriptomic analyses have been deposited in European Nucleotide Archive (ENA) as PRJEB65314. The entire transcriptome is provided in Table S1-8. The sequencing data of phytate enrichments have been deposited in ENA as PRJEB65313. Helius dataset was deposited in European Genome-Phenome Archive (EGA) as EGAS00001002969 (https://ega-archive.org/studies/EGAS00001002969). All raw NMR data are deposited in Figshare. Used databases are biological Magnetic Resonance Data bank (http://www.bmrb.wisc.edu/metabolomics/metabolomics_standards); Pfam (http://pfam.xfam.org/), InterPro (https://www.ebi.ac.uk/interpro/), Brenda (https://www.brenda-enzymes.org/), Uniprot (https://www.uniprot.org/).
