# Supplementary material for: Phytate metabolism is mediated by microbial cross-feeding in the gut microbiota
Source: Nat Microbiol. Author manuscript; Available in PMC 2025 Sep 17. (PMC7618129; doi:10.1038/s41564-024-01698-7)
Supplement: Supplementary information [file EMS207932-supplement-Supplementary_information.docx]

**Table of contents**

- Supplementary figure 1
- Supplementary figure 2
- Supplementary figure 3
- Supplementary figure 4
- Supplementary figure 5
- Supplementary figure 6
- Supplementary figure 7
- MS source parameters and MRM transitions
- Primers used in the mouse work for mouse housekeeping and tight junction genes
- Primers used in Caco-2 cell work for human housekeeping and tight junction genes.


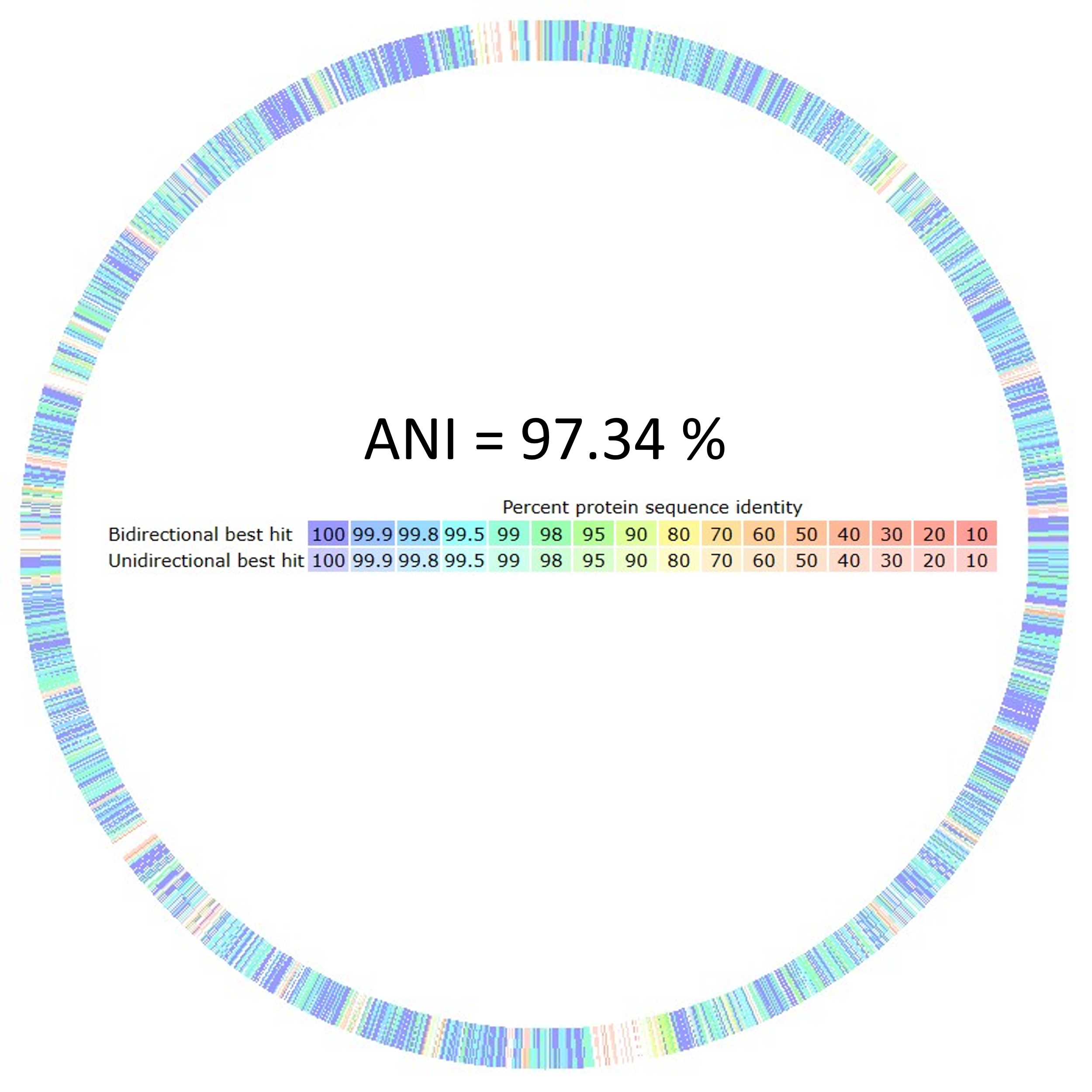


**Supplementary Figure 1**: Genomic comparison between *Mitsuokella* *jalaludinii* H1-1 isolate and *Mitsuokella jalaludinii* DSM13811^T^. Genomic sequence-based comparison between *Mitsuokella* *jalaludinii* H1-1 isolate and *Mitsuokella jalaludinii* DSM13811^T^ isolated from cattle with the average nucleotide identity (ANI) of 97.34%. High sequence homology of the phytate metabolic genes in *Mitsuokella* *jalaludinii* H1-1 isolate compared to that of *Mitsuokella jalaludinii* DSM13811^T^ shown in Table S1. A draft genome of *Mitsuokella jalaludinii* H1-1 isolate has been deposited in the NCBI genome database under BioProject (PRJNA1032471).

**
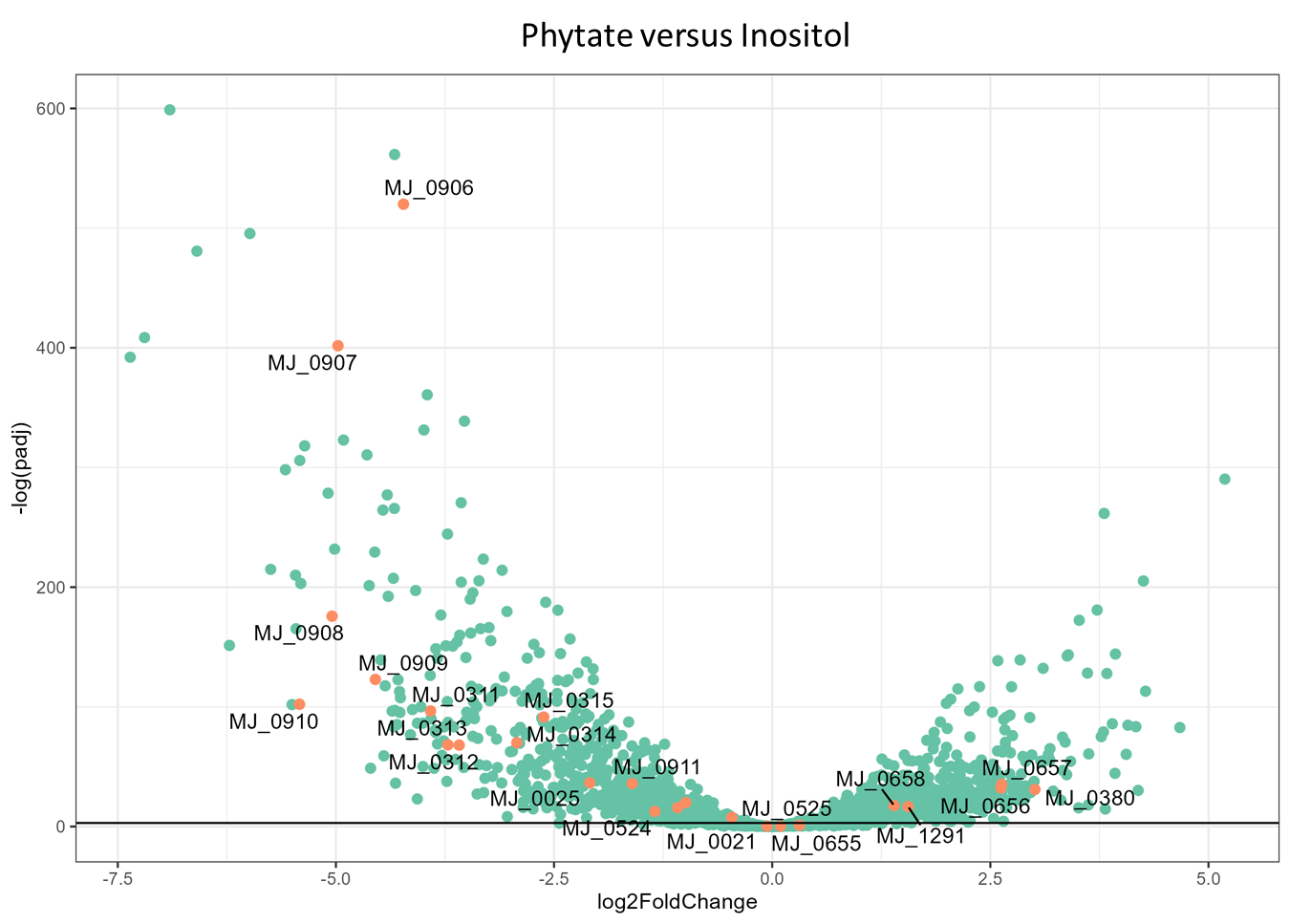
**

**Supplementary Figure 2:** Volcano plot showing differential gene expression of *Mitsuokella jalaludinii* DSM13811^T^ when grown on Phytate as compared to *myo*-Inositol. Genes that are involved in phytate degradation pathway are highlighted and labelled with locus tags. A line indicating the significance threshold (padj=0.05) was added to the figure. Entire transcriptome is provided in Table S2.


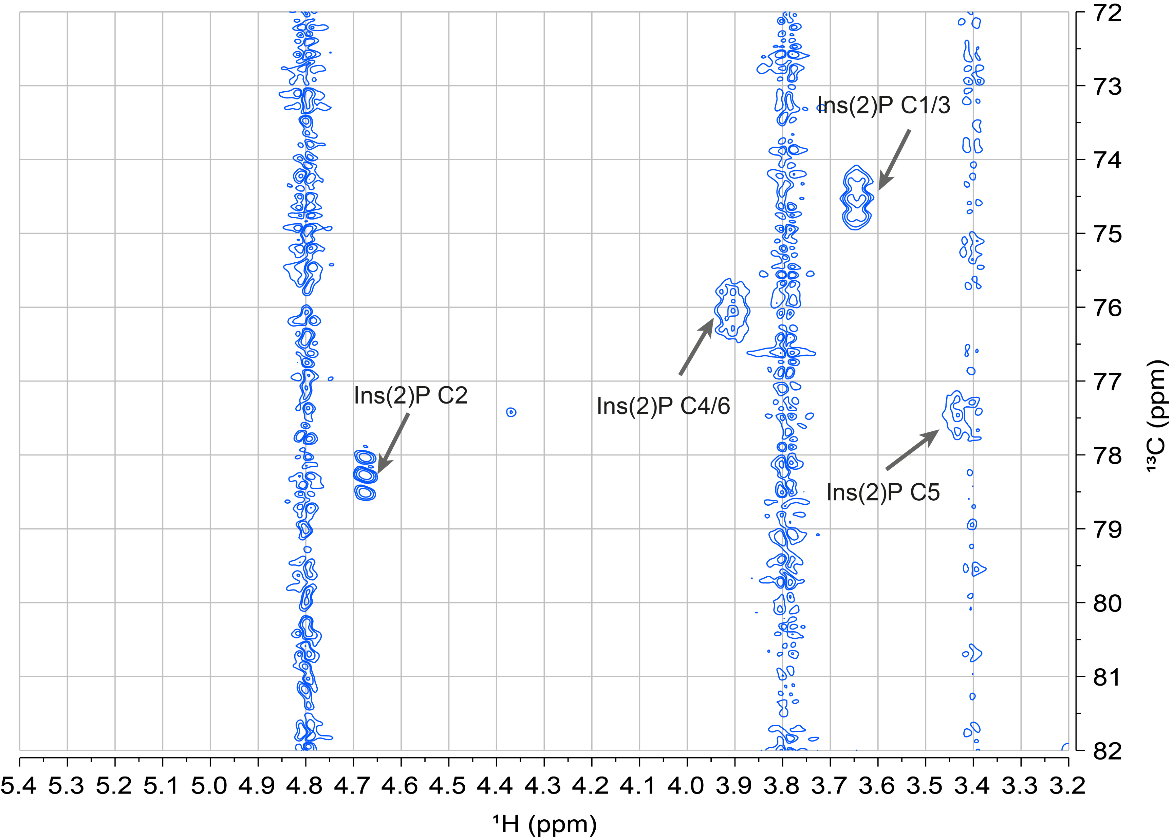


**Supplementary Figure 3**: BIRD-{^1^H,^13^C}HMQC spectrum of the 7.5 h sample of the co-culture of *M. jalaludinii* and *A. rhamnosivorans* in [^13^C_6_]phytate. The region in which inositol polyphosphate signals are commonly found is shown. Similarly, to the monoculture of *M. jalaludinii* (see Fig. 3c), only Ins(2)P was detected as intermediate. Each detectable signal is annotated with the corresponding position of the inositol ring. Note that Ins(2)P exhibits four NMR signals each due to the Cs symmetry of the six-membered inositol ring, and therefore, positions C1 and C3 are magnetically equivalent and so are positions C4 and C6.


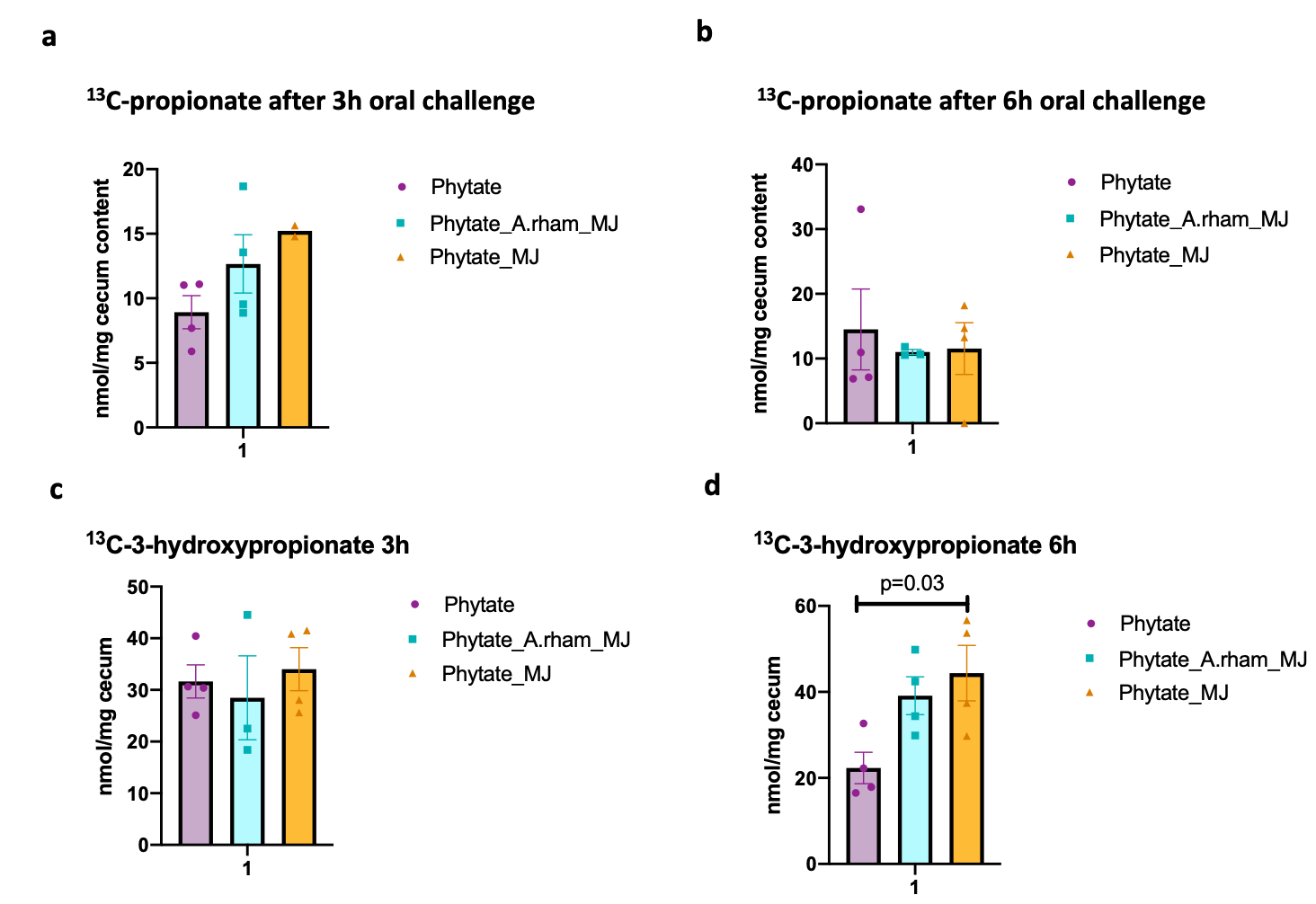


**Supplementary Figure 4:** **Cecal ^13^C-propionate and ^13^C-hydroxypropionate level after 3 h and 6 h oral** oral [^13^C_6_]phytate challenge. Levels of cecal ^13^C-propionate (nmol/mg cecum content) in phytate (purple); Phytate_A.rham_MJ (blue) and Phytate_MJ (orange) treatment groups after 3h (**a**) and 6h (**b**). Shown values are means ± SD (n=4 except Phytate_MJ at 3 h (n=2) and Phytate_A.rham_MJ and Phytate_MJ at 6 h (n=3) due to technical issues during derivatization). Statistical analysis was performed using one-way ANOVA and no significant difference was found. Levels of cecal ^13^C-hydroxypropionate (nmol/mg cecum content) after 3h (**c**) and 6h (**d**). Shown values are means ± SD (n=4). Statistical analysis was performed using one-way ANOVA and only significant difference is indicated. MJ and A.rham_MJ indicate *M. jalaludinii* delivered as monoculture and *A.rhamnisovorans* and *M. jalaludinii* as co-culture, respectively.

**Supplementary Figure 5**: Percentages of five different [^13^C_6_]InsPs in mouse cecum after 6 h oral [^13^C_6_]phytate challenge. Percentages of [^13^C_6_]InsP_6_; [^13^C_6_]InsP_5_[3OH]; [^13^C_6_]Ins(1,2,5,6)P_4_ and InsP_5_[5OH] in mouse ceca of from phytate-only treatment group (purple) Phytate_A.rham_MJ treatment group (blue) and Phytate_MJ (orange) treatment group after 6 h oral [^13^C_6_]phytate challenge. Shown values are means ± SD (n=4) and statistical analysis was performed using one-way ANOVA. Only significantly different comparisons are shown with P values.


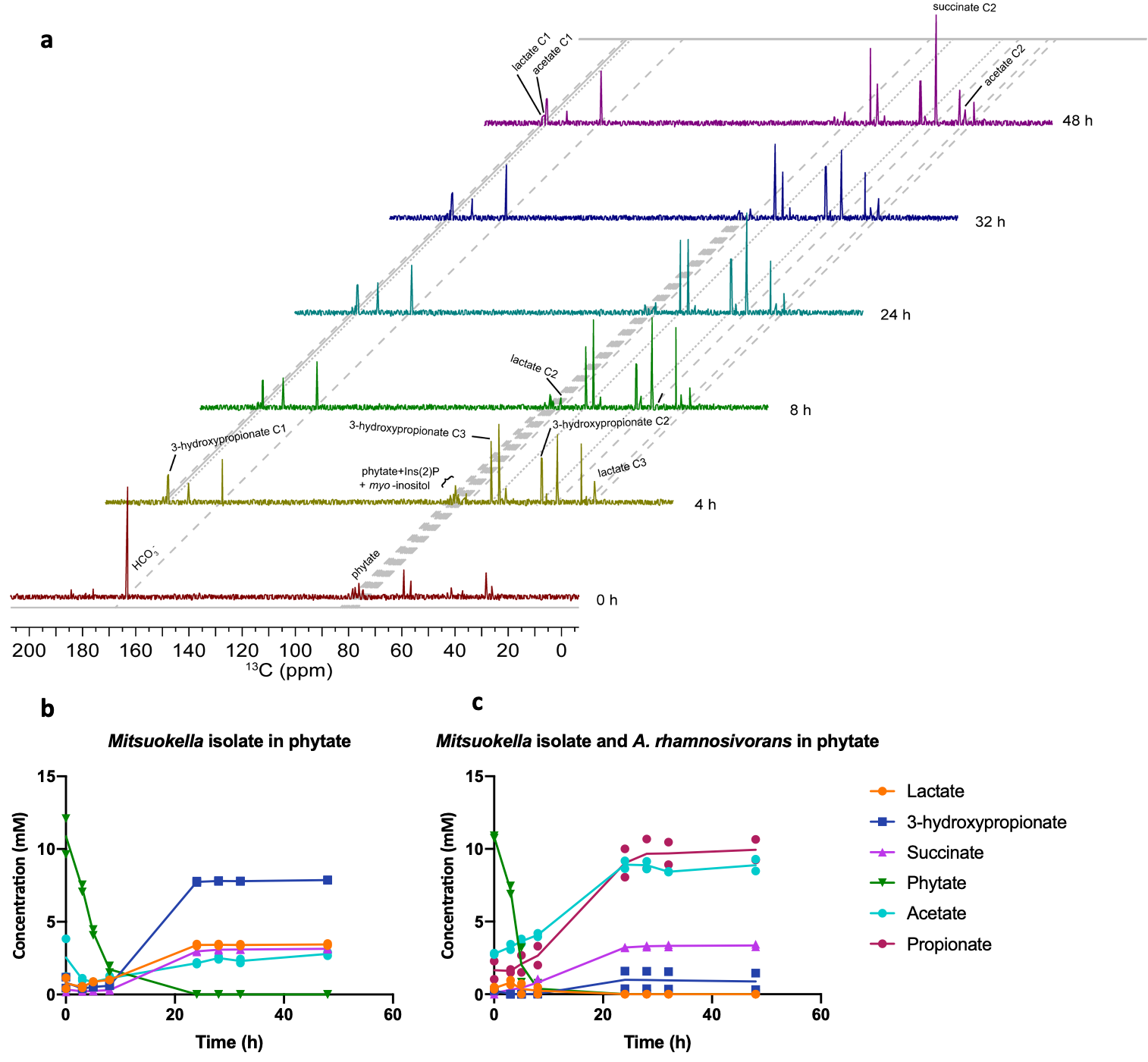


**Supplementary Figure 6**: **Phytate degradation by human *M*. *jalaludinii* strain H1-1 and coculture of *M*. *jalaludinii* H1-1 and *A. rhamnosivorans* in phytate**. **a**. ^13^C-NMR analysis shows rapid [^13^C_6_]phytate degradation with [^13^C_6_]Ins(2)P and [^13^C_6_]Inositol as intermediates and [^13^C_3_]3-hydroxypropionate, [^13^C_3_]lactate, [^13^C_4_]succinate, and [^13^C_2_]acetate as end metabolites. Metabolite production and substrate consumption by monoculture of *M.* *jalaludinii* H1-1 isolate (**b**) and coculture of *M. jalaludinii* H1-1 isolate and *A. rhamnosivorans* (**c**) in phytate (n=2 biological replicates). Data are presented as mean values of two data points.


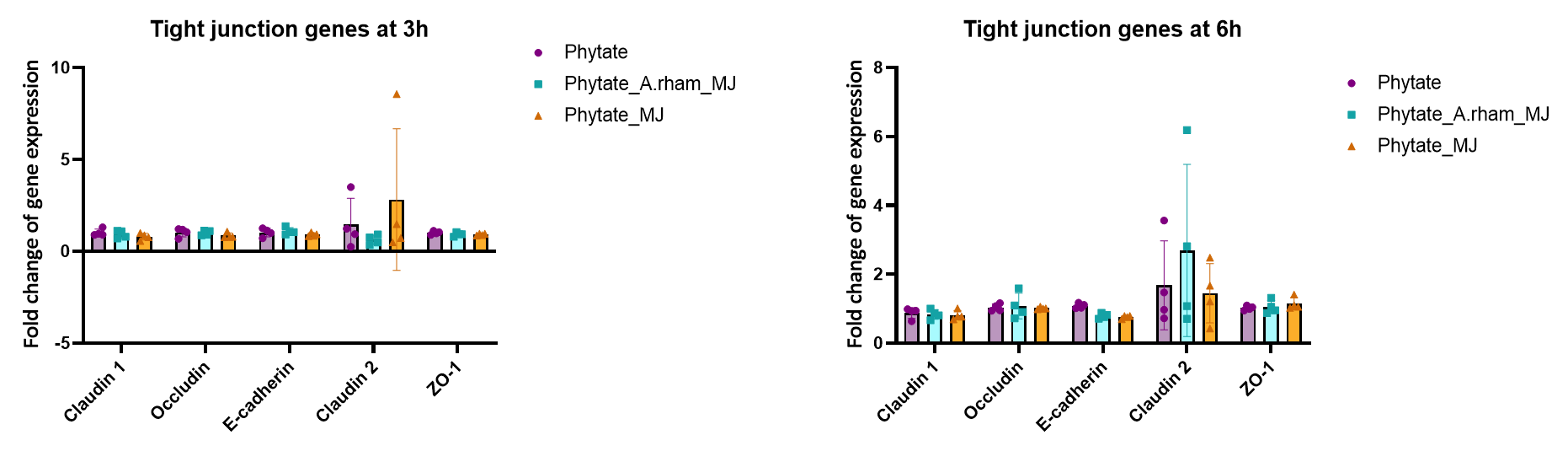


**Supplementary Figure 7**: **Fold change of gene expression of tight junction genes in mouse cecal tissues** in 3 treatment groups. Shown values are means ± SD (n=4 biological replicates) and statistical analysis was performed by two-way ANOVA. No significant difference was observed.

**MS source parameters and MRM transitions**

| **MS Source Parameters** |  |
| --- | --- |
| Gas Temperature | 150 °C |
| Gas Flow | 11 L/min |
| Nebulizer | 8 psi |
| Sheath Gas Temperature | 175 °C |
| Sheath Gas Flow | 8 L/min |
| Capillary Voltage | -2000 V |
| Nozzle Voltage | 2000 V |
| High Pressure RF (Ion Funnel Parameters) | 70 V |
| Low Pressure RF (Ion Funnel Parameters) | 40 V |

| **MRM transitions** | | | | | | | |
| --- | --- | --- | --- | --- | --- | --- | --- |
| Molecular name | Precursor Ion | Product Ion | dwell | Fragmentor (V) | Collision Energy (V) | Cell Accelerator Voltage | Polarity |
| [^13^C_6_] InsP_2_ | 345 | 247 | 50 | 166 | 21 | 4 | Negative |
| [^13^C_6_] InsP_3_ | 424.9 | 326.8 | 50 | 166 | 17 | 4 | Negative |
| [^13^C_6_] InsP_4_ | 252 | 424.9 | 50 | 166 | 5 | 1 | Negative |
| [^13^C_6_] InsP_5_ | 292 | 504.9 | 50 | 166 | 9 | 3 | Negative |
| [^13^C_6_] InsP_6_ | 331.9 | 486.9 | 50 | 166 | 13 | 4 | Negative |
| [^18^O_12_] InsP_6_ | 340.9 | 494.9 | 50 | 166 | 17 | 4 | Negative |
| [^13^C_6_] InsP_7_ | 371.9 | 322.9 | 50 | 166 | 9 | 3 | Negative |
| [^18^O_2_] InsP_7_ | 370.9 | 319.9 | 50 | 166 | 9 | 3 | Negative |
| [^13^C_6_] InsP_8_ | 411.9 | 362.8 | 50 | 166 | 9 | 1 | Negative |

**Primers used in the mouse work for mouse housekeeping and tight junction genes**

|  | Forward (5´🠖 3’) | Reverse (5´🠖 3’) |
| --- | --- | --- |
| m18S | CAC TTT TGG GGC CTT CGT G | GCA AAG GCC CAG AGA CTC ATT |
| m36B4 | GGACCCGAGAAGACCTCCTT | GCACATCACTCAGAATTTCAATGG |
| mZO-1 | TTTTTGACAGGGGGAGTGG | TGCTGCAGAGGTCAAAGTTCAAG |
| mOccludin | ATGTCCGGCCGATGCTCTC | TTTGGCTGCTCTTGGGTCTGTAT |
| mCldn2 | CCAGGGCAATCGTACCAACT | GCCCCTGGTTCTTCACACAT |
| mCdh1 | ATCCTCGCCCTGCTGATTCT | GGCTCTTTGACCACCGTTCT |
| mCldn1 | TGAAGTGCATGAGGTGCCTG | CACTAATGTCGCCAGACCTGAAA |

**Primers used in Caco-2 cell work for human housekeeping and tight junction genes.**

|  | Forward (5´🠖 3’) | Reverse (5´🠖 3’) |
| --- | --- | --- |
| h18S | GAGGGAGCCTGAGAAACGG | GTCGGGAGTGGGTAATTTGC |
| h36B4 | ACGGGTACAAACGAGTCCTG | GCCTTGACCTTTTCAGCAAG |
| hCldn1 | CCTCCTGGGAGTGATAGCAAT | GGCAACTAAAATAGCCAGACCT |
| hOccludin | ATGTCCGGCCGATGCTCTC | TTTGGCTGCTCTTGGGTCTGTAT |
| hCdh1 | GATGCTGATGCCCCCAATACC | GGCCTCAAAATCCAAGCCCTTT |
| hCldn2 | TGGTGCCTGACAGCATGAAA | GGGCTTGGTAGGCATCGTAG |
| hZO-1 | TTCAGAGTGGGGAAACGTCAAT | GCAACTCGGTCATTTTCCTGTA |

Claudin 1 (*Cldn1*), claudin 2 (*Cldn2*), e-cadherin 1 (*Cdh1*), occludin (*Occludin*) and ZO-1 (*ZO-1*)
